# Dietary flavonoid fisetin binds human SUMO1 and blocks sumoylation of p53

**DOI:** 10.1371/journal.pone.0234468

**Published:** 2020-06-12

**Authors:** Vaithish Velazhahan, Przemyslaw Glaza, Alvaro I. Herrera, Om Prakash, Michal Zolkiewski, Brian V. Geisbrecht, Kathrin Schrick

**Affiliations:** 1 Division of Biology, Kansas State University, Manhattan, Kansas, United States of America; 2 Department of Biochemistry and Molecular Biophysics, Kansas State University, Manhattan, Kansas, United States of America; 3 Molecular, Cellular and Developmental Biology, Kansas State University, Manhattan, Kansas, United States of America; Rush University Medical Center, UNITED STATES

## Abstract

Flavonoids are plant-derived compounds that occur abundantly in fruits and vegetables and have been shown to possess potent anti-cancer, antioxidant, and anti-inflammatory properties. However, their direct targets and molecular mechanism of action are not well characterized, hampering exploitation of the beneficial properties of flavonoids for drug development. Small ubiquitin-related modifier 1 (SUMO1) is attached to target proteins as part of a post-translational modification system implicated in a myriad of cellular processes from nuclear trafficking to transcriptional regulation. Using a combination of surface plasmon resonance, differential scanning fluorimetry and fluorescence quenching studies, we provide evidence for direct binding of the dietary flavonoid fisetin to human SUMO1. Our NMR chemical shift perturbation analyses reveal that binding to fisetin involves four conserved amino acid residues (L65, F66, E67, M82) previously shown to be important for conjugation of SUMO1 to target proteins. *In vitro* sumoylation experiments indicate that fisetin blocks sumoylation of tumor suppressor p53, consistent with fisetin negatively affecting post-translational modification and thus the biological activity of p53. A series of differential scanning fluorimetry experiments suggest that high concentrations of fisetin result in destabilization and unfolding of SUMO1, presenting a molecular mechanism by which flavonoid binding affects its activity. Overall, our data establish a novel direct interaction between fisetin and SUMO1, providing a mechanistic explanation for the ability of fisetin to modulate multiple key signaling pathways inside cells.

## Introduction

Flavonoids comprise a family of thousands of closely-related polyphenolic compounds naturally produced by plants. When consumed via the diet, a variety of flavonoids have been shown to possess anti-cancer, anti-oxidant, and anti-inflammatory properties [1, 2]. The dietary flavonoids fisetin and quercetin belong to a subgroup called flavonols which are abundantly found in fruits and vegetables [3]. In particular, fisetin (3,3',4',7-tetrahydroxyflavone) occurs in fruits such as strawberries, apples, and persimmons [4]. The natural properties of flavonoids as a group of compounds have attracted their attention as potential anti-cancer drugs [5]. Fisetin is reported to possess anti-angiogenic and anti-tumor activities in models of human carcinomas [6] and has been shown to inhibit tumor metastasis without exhibiting toxicity to normal cells [7]. A recent study provided evidence for the efficacy of fisetin in combination therapy with paclitaxel (PTX) against A549 non-small cell lung cancer cells [8]. In addition, fisetin is associated with antihyperglycemic, antinephrotoxic, and neuroprotective functions [9–11].

Although much is known about the biosynthetic pathways of flavonoids through a combination of genetic and biochemical approaches [12], direct binding partners and molecular mechanisms underpinning flavonoid action are not well characterized. Many studies have focused on the phenomenological effects on cell lines or animal models without an understanding of the cellular targets of flavonoids. Since the binding mode of flavonoids and key interaction residues in protein targets are poorly understood, drug development efforts incorporating flavonoid mimetics have remained challenging.

Sumoylation of proteins with small ubiquitin-related modifier (SUMO) is a key post-translational modification that regulates fundamental cellular processes such as transcription, intracellular trafficking, and the maintenance of genome integrity [13, 14]. While there are four SUMO isoforms known as SUMO1-4 in humans, *Saccharomyces cerevisiae* has a single SUMO modifier called SMT3 that has been shown to be critical for cell-cycle regulation and chromosome segregation [15–17]. Although SUMO1 and ubiquitin share only ~20% sequence identity, SUMO family members and ubiquitin are highly conserved at the 3D structural level [18]. In addition to the role of sumoylation in response to human pathogens [19], SUMO1 is a well-studied cancer target. Imbalances in sumoylation versus de-sumoylation of oncogenes and tumor suppressors are associated with oncogenic transformation [20]. Cancer targets known to be post-translationally modified by sumoylation include transcriptional regulators such as the tumor suppressor p53 [21], Heat Shock Factor 1 (HSF1) [22], the androgen receptor [23], the c-Jun/AP-1 complex [24], and NF-kappaB [25].

In this study, we investigated the interaction between fisetin and human SUMO1 using a series of binding studies, including surface plasmon resonance (SPR), differential scanning fluorimetry (DSF), and fluorescence quenching. Nuclear magnetic resonance (NMR) experiments were implemented to identify the amino acid residues of SUMO1 involved in binding fisetin. Addressing the biological significance of this interaction, our *in vitro* sumoylation experiments indicate that fisetin interferes with sumoylation of the tumor suppressor protein p53. We propose that the fisetin-SUMO1 interaction has the potential to affect multiple cellular pathways, providing a molecular mechanism underlying the efficacy of flavonoids, for example, in cancer treatments.

## Results

### Fisetin affects electrophoretic mobility of *Saccharomyces cerevisiae* SMT3 and SUMO protease Ulp1

**Fig 1:**
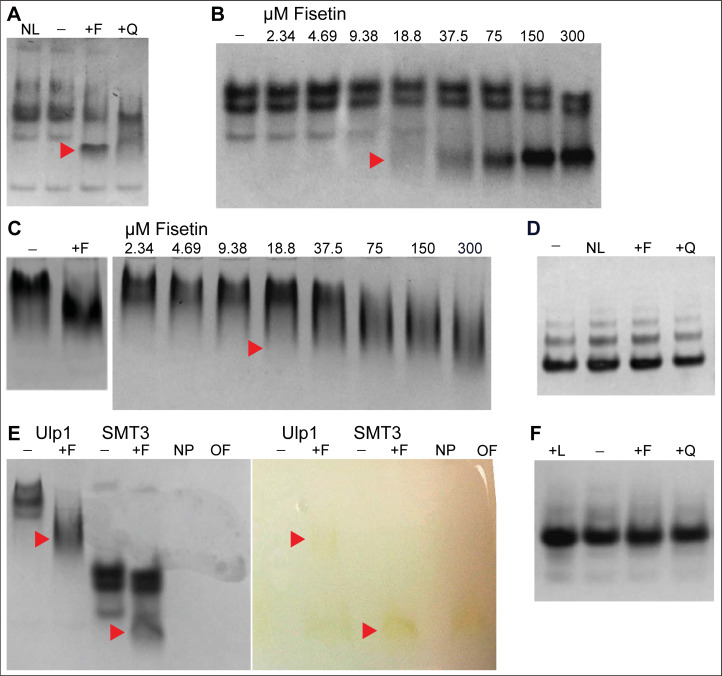
Flavonoids alter mobility of SMT3 and Ulp1 proteins in native-PAGE. (A-F) Coomassie stained Native-PAGE gels; NL, no ligand; −, DMSO; +F, fisetin; +Q, quercetin; +L, luteolin. Concentration of added flavonoid was 300 μM in DMSO unless indicated otherwise. Images are representative of three independent experiments. (A) Fisetin and quercetin cause a shift (red arrowhead) in the migration of SMT3 protein. (B) Fisetin causes a shift (red arrowhead) in the migration of SMT3 (2.75 μM) in a dose-dependent manner. (C) Fisetin causes a shift (red arrowhead) in the migration of Ulp1 (1–2 μM) in a dose-dependent manner. (D) BSA (2 μM) fails to exhibit visible changes in migration upon treatment with fisetin or quercetin. No apparent change in migration of BSA was visible. (E) Gel migration of fisetin, visible by its bright yellow color in matching image (right) of gel prior to Coomassie staining (left), correlates with migration of gel-shifted form of Ulp1 and SMT3 (red arrowheads). NP, no protein or fisetin; OF, only fisetin. (F) No visible change in migration was observed after human SUMO1 (~40 μM) was incubated with the indicated flavonoids (300 μM).

To study interactions between the dietary flavonoid fisetin and specific target proteins, we incubated target proteins that were recombinantly produced in *E*. *coli* with specific flavonoid compounds. In the initial experiments we expressed the human transcription factor HSF1 as a translational fusion to *Saccharomyces cerevisiae* SUMO (SMT3) to increase the solubility of the HSF1 protein. Protein production led to the co-purification of SMT3 along with HSF1 after cleavage with SMT3-specific protease ubiquitin-like-specific protease 1 (Ulp1). Following incubation of fisetin with fractions from the protein mixture containing HSF1, SMT3 and Ulp1, native polyacrylamide gel electrophoresis (native-PAGE) was used to assay for co-migration of interacting molecular species. Surprisingly, the native-PAGE experiments revealed that treatment with fisetin induces a prominent mobility shift in the SMT3 protein (Fig 1A), suggesting molecular interaction concomitant with a change in its hydrodynamic radius. Quercetin, another dietary flavonoid that is structurally similar to fisetin (S1 Fig in S1 Appendix) also affected electrophoretic mobility of SMT3, albeit to a lesser extent (Fig 1A). Fisetin similarly induced a mobility shift when added to the Ulp1 protein. The mobility shift effects occurred in a dose-dependent manner, with band shifts first appearing at ~19 μM fisetin treatment with ~3 μM SMT3 or 2 μM Ulp1, respectively (Fig 1B and 1C).

Since fisetin is reported to bind Bovine Serum Albumin (BSA) [26], we tested whether incubation with fisetin induces a similar electrophoretic mobility shift with BSA. However, in contrast to SMT3 and Ulp1, BSA failed to show a detectable gel shift in our native-PAGE experiments (Fig 1D). Nonetheless, the native-PAGE experiments provided the first evidence for a physical interaction between fisetin and SMT3 as well as for fisetin and Ulp1. In addition to the electrophoretic mobility shift observed for both of these *Saccharomyces cerevisiae* proteins, we visualized co-migration of the intrinsic yellow coloration of fisetin with SMT3 and Ulp1 in native-PAGE (Fig 1E). Since SUMO proteins are highly conserved among the eukaryotes, we next hypothesized that fisetin also interacts with SUMO1, a human orthologue of SMT3. However, we were unable to detect a similar gel shift when recombinantly expressed and purified human SUMO1 was incubated with the dietary flavonoids fisetin, quercetin, or luteolin (Fig 1F). It is noteworthy that treatment of BSA with fisetin also failed to show a change in electrophoretic mobility in our native-PAGE experiments (Fig 1D), despite previously reported evidence for fisetin binding to BSA from multi-spectroscopic studies [26]. Therefore, we proceeded to use additional biophysical techniques to probe whether fisetin directly interacts with human SUMO1.

### Surface plasmon resonance indicates dose-dependent binding of flavonoids to human SUMO1

**Fig 2:**
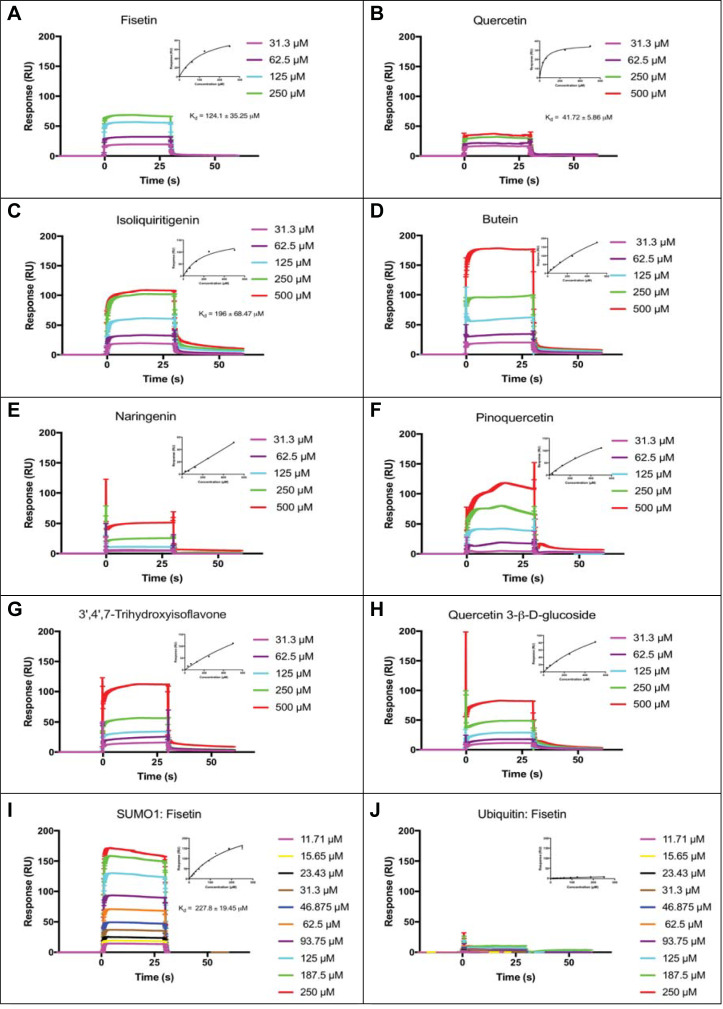
Surface plasmon resonance (SPR) shows dose-dependent binding of human SUMO1 to fisetin, quercetin, and isoliquiritigenin. (A-H) Candidate ligands predicted to bind to recombinant human SUMO1 from the SPR primary screen were tested for dose-dependent binding saturation in a SPR secondary screen. SPR sensograms are shown with fitted curves (insets), performed by fitting resonance units (RU) vs. concentration plots using the classical one-site hyperbola binding equation in GraphPad Prism7. (A) Fisetin, (B) Quercetin, and (C) Isoliquiritigenin exhibited binding that approached saturation with reasonable K_d_ values. (D) Butein, (E) Naringenin, (F) Pinoquercetin, (G) Quercetin 3-β-D-glucoside, and (H) 3’,4’,7-Trihydroxyisoflavone failed to exhibit saturation binding. (I) Binding of fisetin to human SUMO1 was characterized further using an extended range of concentrations, yielding an average K_d_ value of 227.8 ± 19.5 μM. (J) Fisetin failed to bind to bovine ubiquitin. The fitted curve indicates an average of six replicates. Error bars for fitted curves in (I) and (J) correspond to a 95% confidence interval.

We next conducted a surface plasmon resonance (SPR) screen to identify flavonoid binding partners of recombinantly expressed human SUMO1 protein. A binding response threshold of ~15% RU_max_ was used to identify potential flavonoid ligands in the SPR primary screen (Table 1 and S1 Fig in S1 Appendix). Eight compounds that interacted with SUMO1 in the primary screen (fisetin, quercetin, isoliquiritigenin, pinoquercetin, butein, 3’,4’,7-trihydroxyisoflavone, naringenin, quercetin 3-β-D-glucoside) were tested for dose-dependency in secondary SPR screens based on positive sensogram responses and a %RU_max_ above the threshold of ~15% (Table 1, Fig 2, and S2 Fig in S1 Appendix).

**Table 1:** Summary of surface plasmon resonance (SPR) screens and differential scanning fluorimetry (DSF) with SUMO1 to identify flavonoid ligands.

Flavonoid	MW	CAS	%RU_max_	SPR Screens		DSF T_m_ (^o^C)	DSF T_m_ (^o^C)	ΔT (^o^C)
						SUMO1 + DMSO	SUMO1 + Flavonoid	
				1^o^	2^o^			
Fisetin	286.2	345909-34-4	18.9	✓	✓	59.63 ± 0.03	UF	NA
Quercetin	302.24	117-39-5	14.5	✓	✓		UF	NA
Isoliquiritigenin	256.25	961-29-5	73.4	✓	✓		UF	NA
Pinoquercetin	316.26	491-49-6	143.4	✓	×		ND	NA
Butein	272.25	487-52-5	74.2	✓	×		ND	NA
3’,4’,7-Trihydroxy- isoflavone	270.2	485-63-2	48.0	✓	×		33.28 ± 0.15	↓26.35
Naringenin	272.25	67604-48-2	27.5	✓	×		59.24 ± 0.13	↓0.39
Quercetin 3-β-D-glucoside	464.38	482-35-9	21.3	✓	×		56.95 ± 0.28	↓2.68
Quercitrin	448.38	522-12-3	14.2	×	NA		54.28 ± 0.19	↓5.35
Kaempferol	286.24	520-18-3	12.6	×	NA		60.28 ± 0.09	↑0.65
Naringin dihydrochalcone	582.56	18916-17-1	8.5	×	NA		59.94 ± 0.07	↑0.31
Naringin	580.53	10236-47-2	5.4	×	NA		58.93 ± 0.02	↓0.70
C-benzylated chalcone	376.40	102056-84-8	-5.3	×	NA		ND	NA
Luteolin	286.24	491-70-3	-47.8	×	NA		51.89 ± 0.68	↓7.74

Structures of flavonoids are given in S1 Fig in S1 Appendix. %RU_max_ was calculated as (Mean RU/RU_max_)*100, with RU denoting resonance units in the Biacore T200 system. The SPR primary screen was conducted with 500 μM flavonoid in DMSO. A cut-off of %RU_max_ of ~15 was used to select ligands for the SPR secondary screen. Flavonoids that gave negative %RU_max_ values were excluded from the secondary screen. Primary SPR screen: (✓) %RU_max_ with binding activity above cut-off, or (×) low or no binding activity. Secondary SPR screen: (✓) dose-dependent saturation kinetics, or (×) no indication of saturation kinetics. DSF experiments: UF, unfolded protein; ND, melting curve could not be determined; NA, not applicable or not tested; (↑) increase in melting temperature (T_m_); (↓) decrease in T_m_. Mean and SEM are shown (n = 5).

Dose-dependency experiments with the top eight flavonoid candidates from the primary SPR screen (Fig 2A–2H) were consistent with saturable binding for fisetin, quercetin, and isoliquiritigenin, with equilibrium dissociation constant (K_d_) values of ~124 ± 35 μM, ~42 ± 6 μM and ~196 ± 69 μM, respectively (Fig 2A–2C). In an experiment with concentrations of fisetin varying ~20-fold from ~12 to 250 μM, the K_d_ value for binding to SUMO1 was estimated to be ~228 ± 20 μM (Fig 2I). By contrast, we observed no appreciable binding between fisetin and the structurally similar bovine ubiquitin (Fig 2J).

### Fluorescence quenching experiments indicate saturation binding between fisetin and SUMO1

**Fig 3:**
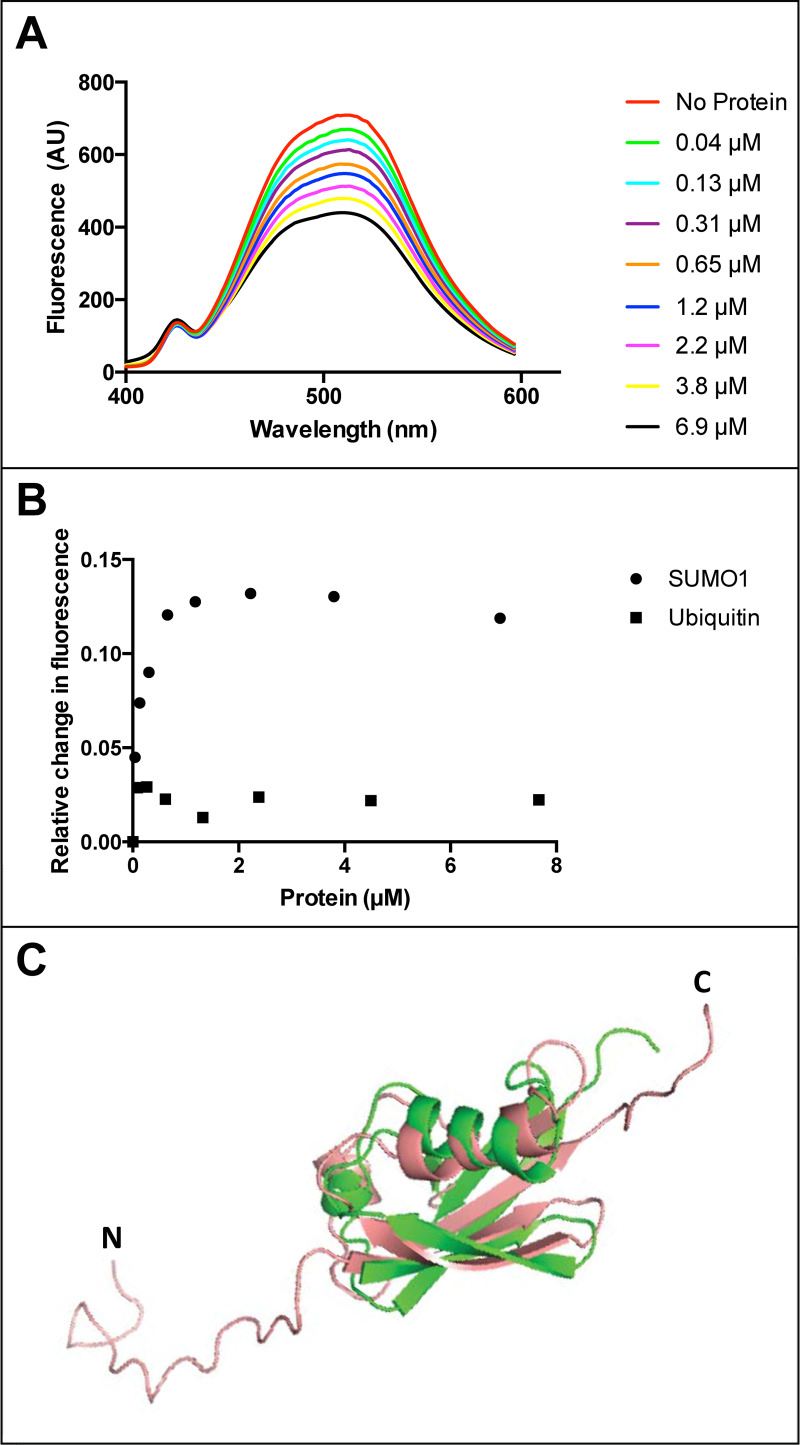
Fluorescence quenching of fisetin with human SUMO1. (A) Fluorescence emission spectra of fisetin indicate quench in fluorescence upon addition of increasing concentrations of purified human SUMO1 protein. (B) Relative change in fluorescence of fisetin (i.e. relative quench of fisetin) after correction for the solvent buffer is plotted against concentration of human SUMO1 (circles) or bovine ubiquitin (squares). For SUMO1, the relative quench of fisetin fluorescence appears saturable. In contrast, ubiquitin addition results in a lower extent of fluorescence quenching. A representative graph is shown from two independent experiments in which a range of SUMO1 and ubiquitin concentrations were tested. (C) Pairwise alignment of SUMO1 (salmon, PDB 1A5R) and ubiquitin (green, PDB 1UBQ) using PyMOL2 illustrates structural 3D-fold conservation despite only ~18% amino acid identity. Amino (N) and carboxyl (C) termini of SUMO1 are indicated.

To independently test for a direct binding interaction between fisetin and human SUMO1 in solution, we investigated quenching of the intrinsic fisetin fluorescence in the presence of SUMO1. The fluorescence spectrum of fisetin was previously reported and used to characterize a binding interaction between fisetin and human serum albumin [27]. We observed that human SUMO1 caused quenching of the fluoresence emission of fisetin in a concentration-dependent manner (Fig 3A). Saturable binding was observed for the interaction between fisetin and human SUMO1, even after the fluorescence emission values were corrected for dilution (Fig 3B). Despite high three-dimensional structural similarity to ubiquitin to SUMO1 (Fig 3C) [18], bovine ubiquitin, when tested over a range of concentrations, showed only marginal quenching of fisetin when the values were corrected for dilution (Fig 3B).

### Differential scanning fluorimetry shows dose-dependent effect of fisetin on the thermal stability of SUMO1

**Fig 4:**
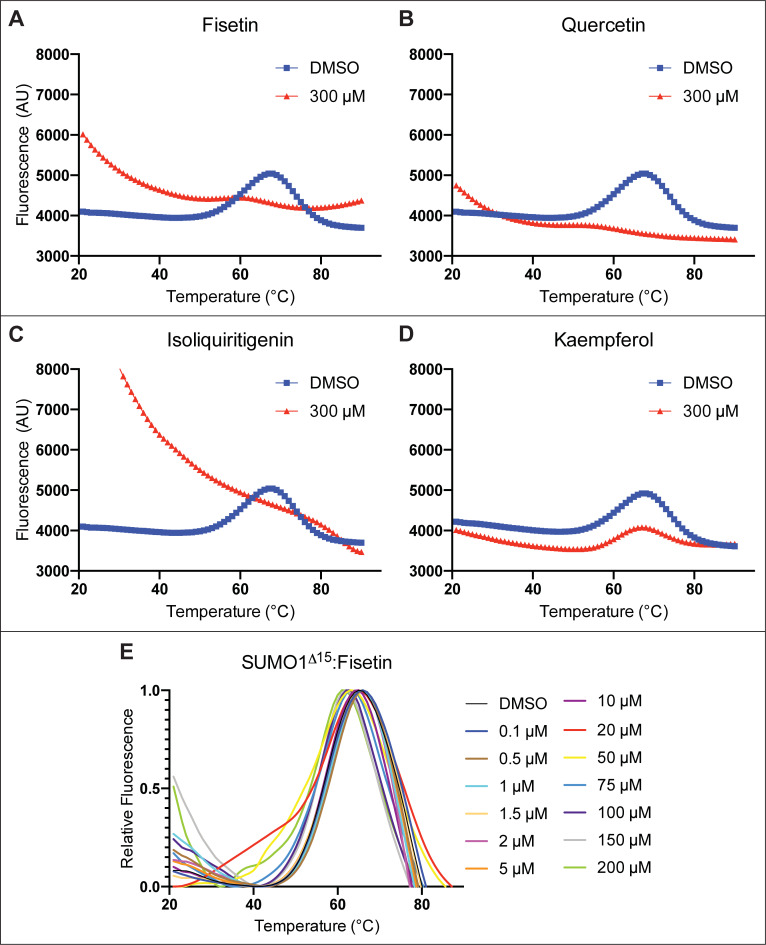
Differential scanning fluorimetry (DSF) shows effect of fisetin and other flavonoids on human SUMO1. Treatment of 2 μM human SUMO1 with 300 μM flavonoid indicated that (A) fisetin, (B) quercetin, and (C) isoliquiritigenin cause unfolding of human SUMO1 leading to a DSF thermogram resembling unfolded protein. (D) In contrast, kaempferol treatment results in little or no effect. (E) Treatment of 2 μM SUMO1^Δ15^ with increasing concentration of fisetin (from 0.1 μM to 200 μM) results in a dose-dependent change in the DSF thermogram of SUMO1. Experiments were performed with five technical replicates.

In thermal shift assays such as differential scanning fluorimetry (DSF), protein-ligand interactions are identified by changes in the thermal stability of a given protein [28]. To assess the thermal stability of human SUMO1 upon interaction with flavonoids, we applied a DSF assay in conjunction with SYPRO^TM^ Orange, a fluorescent dye that binds exposed aromatic residues upon protein unfolding. A DSF thermogram for human SUMO1 exhibited a temperature-induced transition at ~68°C (Fig 4). We next incubated human SUMO1 (2 μM) with an excess (300 μM) of fisetin, quercetin, or isoliquiritigenin, which were the top candidate binding partners identified in the SPR screen (Fig 2 and Table 1). Notably, the resulting DSF thermograms showed a prominent effect on the thermal stability of the SUMO1 protein (Fig 4A–4C). Based on the high fluorescence signal for DSF at 20°C, isoliquiritigenin appears to cause a greater unfolding of SUMO1 as compared to fisetin and quercetin. In contrast, incubation of SUMO1 with excess kaempferol (300 μM) did not appear to greatly alter the thermal stability of SUMO1 in DSF experiments (Fig 4D), suggesting that the effect on SUMO1 depends on the specific type of flavonoid. We also tested the effect of 10 other flavonoids on the DSF thermal stability of SUMO1 (S3A Fig-S3J Fig in S1 Appendix). While a few of the flavonoids displayed little or no effect on thermal stability of the SUMO1 protein, several (such as quercitrin, luteolin, and 3’,4’, 7-tridydroxyisoflavone) resulted in significant thermal destabilization or unfolding of the protein at lower temperatures as compared to the DMSO control (S3H Fig-S3J Fig in S1 Appendix).

One possible explanation for the observed lack of a DSF thermal transition of SUMO1 (Fig 4A–4C) is that high concentrations of fisetin (or quercetin or isoliquiritigenin) cause unfolding of the protein, even at ambient temperature. To compare our results with a known example of protein unfolding, we thermally denatured the human HSF1 protein by heat treatment of 60°C for 15 min to induce protein unfolding. The HSF1 protein is reported to partially unfold during activation [29]. Remarkably, the shape of the resulting DSF thermogram for unfolded HSF1 (S3K Fig in S1 Appendix) resembled that of SUMO1 thermograms upon incubation with 300 μM fisetin, quercetin or isoliquiritigenin (Fig 4A–4C).

**Fig 5:**
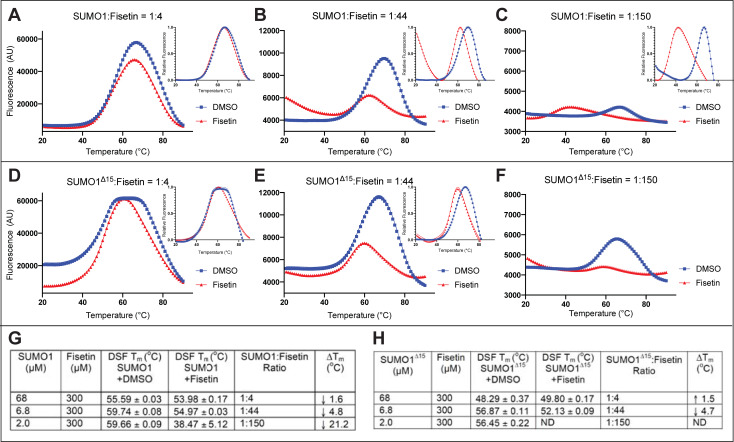
Differential scanning fluorimetry (DSF) reveals concentration-dependent unfolding of SUMO1 and SUMO1^Δ15^ by fisetin. (A-C) Human SUMO1 (68.3 μM, 6.83 μM, or 2 μM) was treated with a DMSO control or fisetin (300 μM in DMSO), giving molar ratios of SUMO1 to fisetin of 1:4, 1:44, and 1:150, respectively. (D-F) The same treatment was performed with SUMO1^Δ15^. (A-E) Relative fluorescence plots are shown in insets. (F) Relative fluorescence was not determined (ND). (G,H) Quantification of DSF data plotted in representative graphs (A-F). As the ratio of SUMO1 to fisetin decreases, increased unfolding is observed for both wild type (SUMO1) and mutant (SUMO1^Δ15^). See Table 1 and S2 Fig in S1 Appendix.

We conducted DSF experiments to measure the dose-dependency of fisetin on the unfolding of SUMO1 (Fig 4E). Here we used a variant of the human SUMO1 called SUMO1^Δ15^ in which the N-terminal 15 amino acids of SUMO1 are removed. The first 15 amino acids of human SUMO1 are weakly conserved and reported to be unstructured [18], and so therefore might contribute to its instability. We nonetheless observed evidence for thermal destabilization of SUMO1^Δ15^ with increasing fisetin concentrations ranging from 20 to 200 μM (Fig 4E). We next compared SUMO1^Δ15^ directly with wild-type SUMO1. A similar response was observed upon treating each protein variant with 300 μM fisetin (Fig 5), indicating that the N-terminal 15 amino acids are not required for the fisetin-SUMO1 interaction. In a series of reciprocal experiments, we set the concentration of fisetin to a constant value of 300 μM and varied the concentration of SUMO1 protein to obtain molar ratios of 1:4, 1:44 and 1:150 (SUMO1:fisetin). Both wild-type SUMO1 and SUMO1^Δ15^ exhibited a molar ratio-dependent unfolding in the presence of fisetin (Fig 5). Taken together, the results indicate that increasing concentrations of fisetin relative to SUMO1 protein result in destabilization and unfolding of the SUMO1 protein.

### NMR chemical shift perturbation analyses of SUMO1 implicate four residues in fisetin interaction

**Fig 6:**
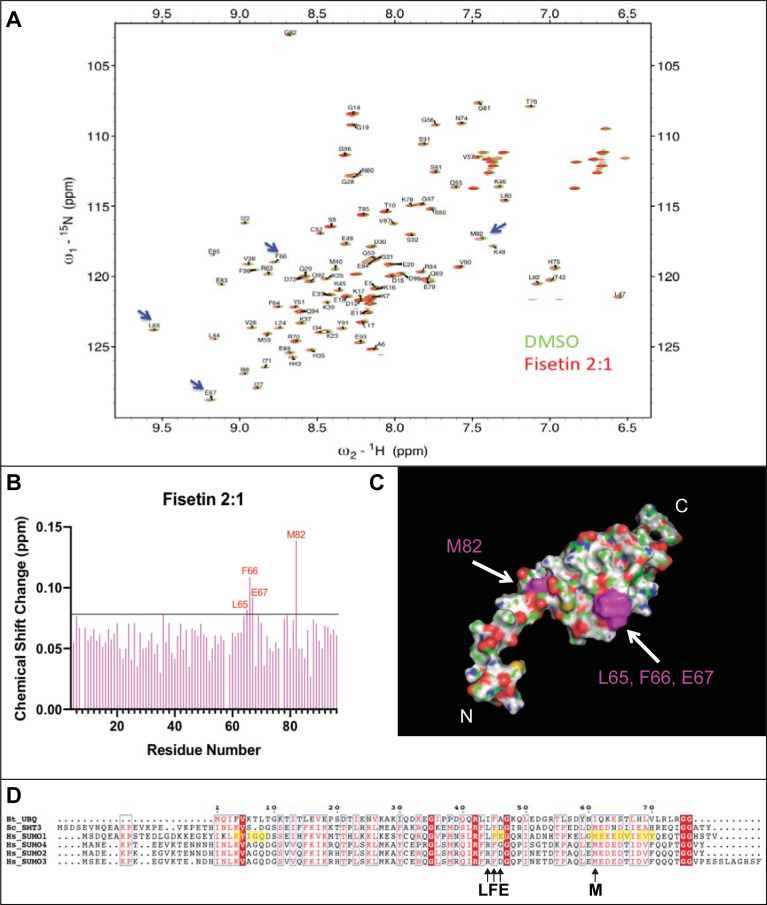
^1^H-^15^N HSQC analyses uncover a chemical shift for four amino acids of SUMO1 on interaction with fisetin. (A) ^1^H-^15^N HSQC spectrum of SUMO1 upon treatment with DMSO vehicle control versus a 2:1 molar ratio of fisetin to protein. Peak assignments and HSQC spectra were rendered using NMRFAM-Sparky [31]. (B) Perturbation analyses of HSQC spectrum show that upon treatment with fisetin, four amino acids of SUMO1 (L65, F66, E67, M82) exhibit a chemical shift change above the cut-off of 0.08. Chemical shift change was calculated as previously described [32]. (C) The four amino acids (L65, F66, E67, M82) (magenta) exhibiting a chemical shift were mapped onto the surface structure of SUMO1 (PDB 1A5R) using PyMOL2. Red, oxygen; blue, nitrogen; green, carbon. (D) ClustalW amino acid alignment of *Homo sapiens* (Hs) SUMO1, SUMO2, SUMO3 and SUMO4 with yeast *Saccharomyces cerevisiae* (Sc) SMT3 and *Bos taurus* (Bt) ubiquitin (UBQ) rendered in ESPript 3.0 [33]. Conserved residues are indicated in red. Note that F66 and M82 are conserved in the SUMO proteins, but not in ubiquitin. SUMO1 residues previously shown to undergo significant chemical shift perturbation upon binding Ubc9 [30] are highlighted.

To identify the residues in human SUMO1 that directly interact with fisetin, we conducted NMR chemical shift perturbation analyses using uniformly ^15^N-labeled SUMO1 protein and performed a series of 2D ^1^H-^15^N HSQC NMR experiments. NMR chemical shift perturbations of backbone amide (^1^HN and ^15^N) resonance in the ^1^H-^15^N HSQC spectra are sensitive probes for changes in chemical environments surrounding amino acid residues. By computing the weighted chemical shift changes of the ^1^H and ^15^N resonances for ^15^N-labeled SUMO1 on binding to fisetin dissolved in DMSO-d_6_, we observed that only four amino acids (L65, F66, E67, and M82) show significant perturbation (>0.08 ppm) when treated with a 2:1 molar ratio of fisetin to SUMO1 (Fig 6A and 6B). Controls using DMSO-d_6_ as a cosolvent caused slight but traceable peak shifts (<0.08 ppm). Mapping of affected residues onto a structural model of SUMO1 (Fig 6C and 6D) revealed the clustering within a conserved region implicated in SUMO E2 conjugating enzyme Ubc-9 mediated conjugation to target proteins [30]. In contrast to fisetin treatment, no significant chemical shift perturbation was observed in 2D ^1^H-^15^N HSQC spectra when ^15^N-labelled SUMO1 was treated with quercetin or kaempferol dissolved in DMSO-d_6_ at a 2:1 molar ratio (S4 Fig in S1 Appendix). In summary, the NMR studies suggest that SUMO1 binding to fisetin involves four conserved amino acid residues (L65, F66, E67, and M82) previously shown to be important for conjugation of SUMO1 to target proteins.

To study dose-dependency of the chemical shift perturbation in the presence of fisetin, we performed NMR titration analyses by treating ^15^N-labeled human SUMO1 (200 μM) with varying concentrations of fisetin (100–450 μM). A significant chemical shift perturbation was observed at a 1:1 molar ratio of fisetin to SUMO1, reaching saturation at higher molar ratios (S5A Fig-S5D Fig in S1 Appendix).

### Fisetin impairs sumoylation of p53 *in vitro*

Having established a direct interaction between fisetin and human SUMO1 in multiple independent biophysical assays, we next investigated the possible functional significance of this interaction. To this end, we performed *in vitro* sumoylation experiments in which the cellular sumoylation reaction was reconstituted using purified human SUMO1, E1 activating enzyme, E2 conjugating enzyme, and two putative protein substrates (human tumor suppressor p53 and human transcription factor HSF1). E1 activating enzyme functions by transferring active SUMO1 protein to the E2 conjugating enzyme Ubc9, which in turn attaches SUMO1 onto target protein substrates [13]. Both p53 and HSF1 proteins are linked to sumoylation as well as to cancer pathways. A previous study revealed that the tumor suppressor p53 is predominantly sumoylated in colon cancer cell lines and that expression of both SUMO1 and p53 leads to increased metastasis [34]. HSF1 is potent driver of malignant transformation [35], and its sumoylation has been shown to stimulate HSF1 DNA binding activity as well as its activation of downstream targets [36].

**Fig 7:**
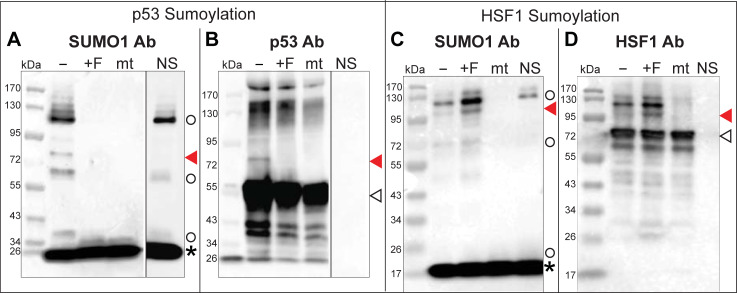
Fisetin blocks *in vitro* sumoylation of p53. *In vitro* sumoylation of (A,B) tumor suppressor protein p53 or (C,D) Heat Shock Factor 1 (HSF1) protein in the absence of (-, DMSO control) or presence of fisetin (+F, 300 μM dissolved in DMSO). Western blots were probed with (A,C) SUMO1, (B) p53 or (D) HSF1 antibody (Ab) as indicated. Sumoylated substrate proteins (red arrowheads), unmodified substrate proteins (open arrowheads), E1 or E2 SUMO1 conjugates (open circles) and SUMO1 (asterisk) are indicated. A SUMO1 conjugation-deficient mutant (mt) served as a negative control; NS, a no substrate control allows detection of E1 and E2-SUMO conjugates with the SUMO1 Ab. (A,B) The p53 substrate is visibly sumoylated only when fisetin is absent. No E1 or E2 conjugates are detected in the presence of fisetin. A dividing line between lanes indicates that the NS controls are from separate blots. (C,D) The HSF1 substrate is sumoylated in both the absence and presence of 300 μM fisetin. Western blots are representative of at least two independent experiments.

Using the tumor suppressor p53 protein as a putative substrate, we observed that a band corresponding to sumoylated p53 appeared as expected upon treatment with the DMSO control (Fig 7A and 7B). The higher MW species (~110–120 kDa) appearing in SUMO1 antibody Western blots represents E1 and E2 SUMO conjugates that are intermediates in the sumoylation reaction and these conjugates were also detected when no p53 substrate is included (Fig 7A). Strikingly, when the sumoylation reaction was carried out in presence of 300 μM fisetin, no SUMO conjugates of p53 were detected. Notably, E1 and E2 SUMO conjugates were not detected either (Fig 7A). To determine whether fisetin globally blocks sumoylation of other targets, we examined sumoylation of another putative protein substrate, recombinantly produced HSF1, in the presence of 300 μM fisetin. In contrast to the inhibition of sumoylation of tumor suppressor protein p53, fisetin appeared to slightly enhance E1/E2 SUMO conjugates and sumoylation of HSF1 (Fig 7C and 7D).

## Discussion

### Multiple biophysical assays provide evidence for fisetin-SUMO1 interaction

In this study we provide multiple lines of evidence for a direct interaction between the dietary flavonoid fisetin and the human SUMO1 protein. SPR analyses confirmed dose-dependent binding of fisetin to SUMO1 with an estimated K_d_ ranging from 124 to 228 μM (Fig 2A and 2I). In contrast to SPR, the fluorescence quenching, NMR HSQC and thermal shift DSF experiments probed the interaction of fisetin with SUMO1 in solution. While we were unable to estimate a K_d_ value from the fluorescence quenching studies due to the unknown binding stoichiometry of SUMO1 to fisetin under the assay conditions, the data are consistent with saturable binding (Fig 3A and 3B).

The binding studies additionally suggest that the molecular interaction of fisetin with human SUMO1 exhibits specificity. Although the SPR experiments indicated quercetin binding to SUMO with a K_d_ of ~42 μM (Fig 2B), in HSQC perturbation analyses, quercetin failed to show a chemical shift change above the cut-off value (S4A Fig, S4B Fig in S1 Appendix). The structures of fisetin (3,3′,4′,7-tetrahydroxyflavone) and quercetin (3,3',4',5,7-pentahydroxyflavone) differ by only one extra hydroxyl group in quercetin, whereas kaempferol (3,4′,5,7-tetrahydroxyflavone) differs from fisetin only in the position of its hydroxyl group (S1 Fig in S1 Appendix). Despite the high similarity in the structures of these flavonoids, kaempferol seems to show only weak interaction with human SUMO1, as assayed by either SPR or NMR HSQC perturbation analyses (Table 1 and S4C Fig, S4D Fig in S1 Appendix).

Thermal shift assays using DSF provided further insight on the fisetin:SUMO1 interaction. Protein thermal stability is predicted to be affected by molecular interactions, and thus a change in melting temperature (ΔT_m_) in the presence of a ligand is often an indication of a direct interaction (Niesen et al., 2007). Collectively, the DSF results demonstrate that fisetin affects the thermal stability of SUMO1 in a dose-dependent manner (Figs 4 and 5). While high concentrations of fisetin (300 μM fisetin: 2 μM SUMO1; corresponding to a 150:1 molar ratio) result in unfolding of the protein at ambient temperature, molar ratios of 4:1 give only slightly affect thermal stability, as estimated by ΔT_m_ less than 2°C (Fig 5). Thus, the observed unfolding of SUMO1 appears to be dependent upon high concentrations of fisetin relative to protein. It is noteworthy that the NMR HSQC perturbation analyses did not detect unfolding of the SUMO1 protein in the presence of fisetin (Fig 6 and S5 Fig in S1 Appendix). The NMR experiments required a high concentration of SUMO1 protein (200 μM) in conjunction with a 100–450 μM range of concentrations of the flavonoid and thus the molar ratio of fisetin:SUMO in these experiments was low (ranging from 0.5:1.0 to 1.5:1.0). Considering that the DSF experiments show SUMO1 protein unfolding as the flavonoid to protein ratio exceeds ~150:1.0 (Figs 4 and 5), it is not surprising that we did not detect large perturbations in the NMR ^1^H-^15^N HSQC studies in which the flavonoid:protein molar ratio was 100-fold lower (1.5:1:0) (Fig 6 and S5 Fig in S1 Appendix).

### Molecular identification of amino acids implicated in fisetin-SUMO1 interaction and effect of fisetin on sumoylation activity

From the NMR HSQC perturbation analyses of SUMO1, only four amino acids (L65, F66, E67, M82) undergo a significant chemical shift with a 2:1 molar ratio of fisetin to protein (Fig 6A and 6B). The amino acids F66 and M82 are conserved among the SUMO family members (Fig 6D). Our results indicate that fisetin fails to interact with bovine ubiquitin in both SPR (Fig 2J) and fluorescence quenching (Fig 3B) assays despite a highly similar 3D structure (Fig 3C). Sequence alignment of ubiquitin with *Saccharomyces cerevisiae* SMT3 and human SUMO1-4 reveals that only one of the four residues involved in fisetin interaction (F66) is conserved in ubiquitin (Fig 6D). The presence of a negatively charged residue at position 67 (conserved in SUMO1-3) and a methionine at position 82, which shows the highest chemical shift change upon fisetin binding (Fig 6A), could be important for the observed interaction. Amino acids F66, E67 and M82 map to a region of conserved residues shown to be important for Ubc9 conjugation to SUMO1 [30] (Fig 6D). By interacting with SUMO1, fisetin could preclude interaction with the E2 conjugating enzyme Ubc9, thus interfering with SUMO conjugation to target proteins.

To test this idea, we performed *in vitro* sumoylation studies in which we reconstituted physiological sumoylation reaction using purified components. Our results indicate that fisetin completely inhibits sumoylation of the tumor supressor p53 (Fig 7). It is notable that not even E1/E2-SUMO conjugates, which are intermediates in the sumoylation reaction, were detected in the presence of fisetin. Given the strong effect on p53 sumoylation, it is puzzling that fisetin treatment failed to alter sumoylation of another substrate, namely the human transcription factor HSF1. Perhaps fisetin does not globally disrupt SUMO conjugation, but instead exerts differential effects on SUMO activity that are dependent on affinity to the target substrate. One possibility is that molecular interactions of fisetin with HSF1 interfere with or alter the SUMO1-fisetin interaction when all three components are combined *in vitro*. In the case of HSF1, it has been shown that sumoylation of this transcription factor stimulates its DNA binding activity [36]. Moreover, it is reported that fisetin disrupts the DNA binding activity of HSF1 *in vivo* [37]. Cancer cells exhibit high expression of HSF1 [38], and *in vivo* sumoylation of HSF1 by SUMO1 is dependent upon phosphorylation of a specific serine residue [39]. While beyond the scope of the present study, it would be interesting to examine the effect of fisetin treatment on the sumoylation of HSF1 in cancer cells.

### Connection between the fisetin-SUMO1 interaction, p53 and other cancer pathways

Fisetin was previously reported to induce the p53-mediated apoptotic pathway in human renal carcinoma cells [40]. Yet, the underlying mechanism and direct connection to SUMO1 has not been described to date. Numerous studies indicate that sumoylation is elevated in tumor cells in comparison to normal cells [41]. In colon cancer patients, it was shown that the combined elevated expression of SUMO1 and p53 leads to increased metastasis, and moreover, p53 is predominantly sumoylated in colon cancer cell lines [34]. Fisetin has also been shown to exert its anti-cancer effect by inducing cell cycle arrest and apoptosis in bladder cancer cells through activation of p53 [42]. Our study demonstrates that fisetin treatment inhibits p53 sumoylation, suggesting a molecular mechanism underlying fisetin action. It was previously shown that p53 sumoylation promotes nuclear export and thus inactivation of p53-mediated apoptosis [43]. Overall, our findings suggest a potential use for fisetin and its mimetics as novel p53 sumoylation inhibitors in cancer treatment.

Fisetin has been reported to impact other disease pathways, such as those mediated by HSF1 [37], NF-kappaB [44] and c-Jun/AP-1 [45], each of which has established connections to both cancer and sumoylation. We propose that direct interaction between fisetin and SUMO1 could provide an explanation for fisetin action and modulation of key pathways involved in tumor progression and metastasis. The propensity for this interaction seems to be evolutionarily conserved, since we detected a fisetin-induced mobility shift for SUMO1 ortholog SMT3 and SUMO-specific protease Ulp1 from yeast in native-PAGE experiments (Fig 1). Our finding that fisetin interacts with *Saccharomyces cerevisiae* SUMO-specific protease Ulp1 (Fig 1B) suggests that in a cellular context the effect of fisetin could be dependent on both its interaction with SUMO1 and the corresponding SUMO-specific protease. Since mammalian SUMO deconjugating enzyme SENP1 bears substantial similarity to yeast Ulp1 [46], fisetin interaction with SUMO deconjugating enzyme could also dictate the physiological consequence of fisetin interaction in sumoylation pathway in a cellular context. Further experiments need to be conducted to validate direct interaction between human SENP1 and fisetin and explain how SENP1-fisetin and SUMO1-fisetin interactions lead to differential sumoylation outcomes. Hence, the novel SUMO1-fisetin interaction and its downstream ramifications await further investigation to reveal the mechanistic basis of flavonoid action in cancer cells and could aid future drug development efforts.

## Materials and methods

### Flavonoids and native bovine ubiquitin

Fisetin (F4043), kaempferol (K0133), luteolin (L9283), naringenin (W530098), naringin (N1376), quercetin (Q4951), quercetin 3-β-D-glucoside (17793), and quercitrin (Q3001), and native bovine ubiquitin (U6253) were obtained from Sigma-Aldrich (St. Louis, MO). C-benzylated chalcone (NP-000319) and pinoquercetin (NP-012356) were obtained from AnalytiCon Discovery (Potsdam, Germany). Butein (W1236) and isoliquiritigenin (0271) were obtained from AK Scientific Inc. (Union City, CA). 3’,4’,7-Trihydroxyisoflavone (20930) was obtained from Cayman Chemical Co. (Ann Arbor, MI). Flavonoids were dissolved in DMSO or another solvent, according to application as described below, and stock solutions were stored at -20°C. Bovine ubiquitin was dissolved in a buffer according to assay (see below).

### Recombinant expression and purification of Saccharomyces cerevisiae SMT3 and HSF1

*Saccharomyces cerevisiae SMT3* was expressed as a translational fusion with human Heat Shock Factor 1 (HSF1). The plasmid encoding 6xHis:SUMO(SMT3):HSF1 was a gift from Matthias Mayer. The protocol for expression and purification of HSF1 was adapted from [29]. Primary cultures were started by inoculating a colony of freshly transformed BL21 Rosetta (DE3) (Novagen, MilliporeSigma, Burlington, MA) cells into 50 ml 2xYT media grown at 37°C for 12–16 hr. Secondary cultures were inoculated at 1:500, grown at 37°C to OD_600_ of 0.6–0.7, and was shifted to 20°C before induction with 0.1 mM IPTG. The induced culture was grown for 2 hr at 20°C, and the cells were collected by centrifugation and frozen at −80°C until use. Cell pellets were resuspended in lysis buffer (25 mM Hepes pH 7.4, 150 mM NaCl, 10% glycerol, 3 mM β-mercaptoethanol, 25 units/μl benzonase, 1% Pierce Protease Cocktail Inhibitor (EDTA-free), 1 mg/ml lysozyme, 5 mM imidazole, 1 mM PMSF). Cells were lysed twice using a French press at 700 psi, followed by centrifugation at high speed for 1 hr to remove cell debris. The supernatant containing 6xHis-SUMO-HSF1 was incubated for 1 hr with 6 ml TALON resin (Clontech, Mountain View, CA) and was centrifuged at 700xg for 5 min. The supernatant was cleared, and the resin containing the bound target protein was transferred to a gravity flow column, washed with 3–5 column volumes of wash buffer (25 mM Hepes pH 7.4, 150 mM NaCl, 10% glycerol, 3 mM β-mercaptoethanol, 5 mM imidazole) until the flow-through showed baseline absorption at A595 with Bradford reagent. The 6xHis-SUMO-HSF1 protein was eluted in 25 mM Hepes pH 7.4, 150 mM NaCl, 10% glycerol, 3 mM β-mercaptoethanol, and 250 mM imidazole. Protein fractions were monitored on SDS-PAGE to assess purity. To separate SMT3 from HSF1, the eluted fractions containing the target protein were cleaved with recombinantly produced *Saccharomyces cerevisiae* Ulp1 at 4°C for 8–12 hr. Cleaved protein was dialyzed with 25 mM Hepes pH 7.4, 150 mM NaCl, 10% glycerol, 3 mM β-mercaptoethanol, and 5 mM imidazole, and the Ulp1 and SMT3 containing 6X His tag remained bound to the TALON resin. Ulp1 and SMT3 were separated and further purified by size exclusion chromatography using Superdex 75 (GE Healthcare, Chicago, IL). Purity was monitored on SDS-PAGE. Fractions containing HSF1 after cleavage were purified by size exclusion chromatography using Superdex 200 (GE Healthcare, Chicago, IL) and fractions were similarly monitored on SDS-PAGE for purity (S6 Fig in S1 Appendix).

### Recombinant expression and purification of Saccharomyces cerevisiae Ulp1

The plasmid encoding 6xHis-Ulp1 was a gift from Matthias Mayer. Primary cultures were started by inoculating a single colony of freshly transformed BL21 (DE3) (Novagen, MilliporeSigma, Burlington, MA) cells into 50 ml 2xYT supplemented with 2 mM MgSO_4_, and grown at 30°C for 12–16 hr. Secondary cultures were inoculated in 2xYT media supplemented with 2 mM MgSO_4_ at 1:500 dilution, grown at 30°C to an OD_600_ of 1, and shifted to 20°C for 1 hr before induction with 0.5 mM IPTG. The induced culture was grown overnight at 20°C, and cells were collected by centrifugation at 4°C, and frozen at −80°C until use. Cell pellets were resuspended in lysis buffer (40 mM Hepes, KOH pH 7.4, 150 mM KCl, 20 mM β-mercaptoethanol, 25 units/μL benzonase, 1% Pierce Protease Cocktail Inhibitor (EDTA-free), 1 mg/mL lysozyme, 5 mM imidazole, 1 mM PMSF). Cell pellets were sonicated for 6–8 min with 20 sec 'on' and 40 sec 'off' cycles. The disrupted lysate was clarified by centrifugation at high speed for 1 hr. Supernatant containing the 6xHis-SUMO-HSF1 protein was incubated for 1 hr with 6 ml TALON resin, followed by centrifugation at 700xg for 5 min. The supernatant was cleared, and the resin containing the bound target protein was transferred to a gravity flow column, washed with 3–5 column volumes of wash buffer (40 mM Hepes, KOH pH 7.4, 150 mM KCl, 20 mM β-mercaptoethanol, 5 mM imidazole) until the flow-through showed baseline absorption at A595 with Bradford reagent. The 6xHis-Ulp1 protein bound to TALON resin was eluted with 40 mM Hepes, KOH pH 7.4, 150 mM KCl, 3 mM β-mercaptoethanol, and 250 mM imidazole. Protein fractions were monitored on SDS-PAGE to assess purity. The activity of purified Ulp1 was verified by cleavage of the 6xHis:SUMO(SMT3):HSF1 substrate with maximum cleavage observed at a 1:100 dilution.

### Recombinant expression and purification of human SUMO1 and SUMO1^Δ15^

The pET11a-SUMO1 plasmid encoding amino acids 1–97 of mature human SUMO1 (referred herein as wild-type SUMO1) was acquired from Addgene (#53138). The protocol for recombinantly expressing and purifying human SUMO1 was adapted from [47]. The plasmid encoding SUMO1^Δ15^ (in which N-terminal 15 amino acids of SUMO1 was deleted leaving the initiating methionine) was constructed from pET11a-SUMO1 with Q5 Site-Directed Mutagenesis Kit (New England Biolabs, Ipswich, MA) using the oligonucleotides 5’-AAGGAAGGTGAATATATTAAACTC-3’ and 5’-CATATGTATATCTCCTTCTTAAAG-3’. Primary cultures were started by inoculating a colony of freshly transformed BL21 Rosetta (DE3) (Novagen, MilliporeSigma, Burlington, MA) cells into 50 ml LB media supplemented with 1 mM MgCl_2_ and 0.1% glucose followed by growth at 37°C for 12 hr. Secondary cultures were inoculated at 1:500 dilution and grown at 37°C to OD_600_ of 0.6. Cultures were induced with 1 mM IPTG and incubated for 4–6 hr at 37°C. Cells were collected by centrifugation and frozen at −80°C. Cell pellets were resuspended in lysis buffer (50 mM Tris-HCl pH 8.0, 50 mM NaCl, 25 units/μL benzonase, 1% Pierce Protease Cocktail Inhibitor (EDTA-free), 1 mM DTT, 1 mM PMSF). Lysis was performed by passing twice through a French press at 700 psi, followed by centrifugation at high speed for 1 hr. The supernatant was incubated at 4°C for 2 hr with 10 ml Q-sepharose equilibrated with lysis buffer containing 50 mM Tris-HCl pH 8.0, 50 mM NaCl, and 1 mM DTT, followed by centrifugation at 250xg for 15 min. The supernatant containing the target protein was concentrated using 3-kDa MWCO spin columns. Low levels of insoluble protein were removed using a 0.45 μm low-protein binding filter. The protein was further purified by size exclusion chromatography using Superdex 75 (GE Healthcare, Chicago, IL) equilibrated in 1X PBS buffer. Purity of the fractions was monitored by SDS-PAGE. See S7A Fig, S7B Fig in S1 Appendix.

### Preparation of ^15^N-labeled human SUMO1

A colony of pET11a-SUMO1 freshly transformed BL21 Rosetta (DE3) (Novagen, MilliporeSigma, Burlington, MA) cells was inoculated into 50 ml LB media supplemented with 1 mM MgCl_2_ and 0.1% glucose, and grown at 37°C for 14 hr. Cells from this primary culture were collected by centrifugation at 4°C and resuspended in 1 L M9 minimal media containing 6g Na_2_HPO_4_, 3g KH_2_PO_4_, 0.5 g NaCl, 1g ^15^N NH_4_Cl (NLM-467-PK, Cambridge Isotope Laboratories, Inc.) except that choline, pantothenate, nicotinamide and pyridoxal components were used instead of biotin. Secondary cultures were grown at 37°C to OD_600_ of 0.6, and induced with 1 mM IPTG for 4–6 hr at 37°C. Cells were harvested by centrifugation at 4°C, and pellets were frozen at −80°C until use. ^15^N-labeled SUMO1 was purified from cell pellets as described above. See S7 Fig in S1 Appendix.

### Fluorescence quenching studies with fisetin

A fisetin stock solution (7 μM) was prepared by dissolving fisetin in mass spectrometry grade methanol. The working solution of 0.5 μM fisetin was prepared by serial dilution in 50 mM Hepes buffer. The spectrum between 390–600 nm was measured with a PerkinElmer LS-55 fluorescence spectrometer. Excitation for fisetin was 390 nm and two emission peaks were observed at 425 and 511.5 nm, respectively. The peak at 511.5 nm was chosen as reference to calculate quench in the observed fluorescence intensity of fisetin. Excitation and emission slit wavelengths were 5 and 10 nm, respectively, with scan speed of 500 nm/min. Ten replicate scans in cumulative capture mode were collected for fisetin without protein and for each concentration of added protein. Binding of human SUMO1 to fisetin was studied in 50 mM Hepes, pH 7.4 and in 20 mM Tris-Cl pH 7.0. Binding studies with native bovine ubiquitin to fisetin were performed in 50 mM Hepes buffer, pH 7.4. Values were corrected for dilution as follows: Relative fluorescence values were calculated by subtracting fluorescence quenching observed upon addition of buffer alone.

### Surface plasmon resonance

All injections were performed in a Biacore T200 (GE Healthcare, Chicago, IL) at a flow rate of 20 μl/min in running buffer (20 mM Hepes (pH 7.4), 150 mM NaCl, 5 mM MgCl_2_, 0.0005% Tween 20) at 25°C. CMD200 and HC1500 sensor chips (Zantec Bioanalytics, Duesseldorf, Germany) were used for the primary and secondary SPR screens and for obtaining SPR sensograms shown in Fig 2I and 2J, respectively. SUMO1 surface in CMD-200 sensor chip and HC1500 sensor chip were generated with immobilization levels 10887 and 19639 resonance units (RU), respectively. Ubiquitin surface in HC1500 sensor chip was generated with 6106 RU. Human SUMO1 and bovine ubiquitin biosensors were prepared by standard amine coupling chemistry as follows: An equal volume of 0.1 M N-hydroxysuccinimide (NHS) and 0.4 M ethyl(dimethylaminopropyl) carbodiimide was injected for 7 min at 5 μl/min over a single flow cell. Following this step, human SUMO1 or bovine ubiquitin (100 μg/ml in pH 4.5 acetate buffer) was injected for 15 min, immediately followed by a 7 min injection of 1M ethanolamine (pH 9). Solvent correction curves were generated at the beginning, end, and after every 50 injection cycles with varying DMSO (control) concentrations (4.0, 4.4, 4.6, 4.8, 5.0, 5.2, 5.4, and 5.9% [v/v]). Ethyl(dimethylaminopropyl) carbodiimide/NHS activation followed by immediate ethanolamine quenching was used to generate a reference flow cell for each sensor chip. For the primary SPR screen, flavonoids (500 μM dissolved in DMSO) were injected for 2 min followed by a 1 min dissociation phase. For the secondary SPR screen, five concentrations (500 μM, 250 μM, 125 μM, 62.5 μM, 31.25 μM) of flavonoid were injected for 2 min followed by a 1 min dissociation phase. RU_max_ was calculated as 8320/MW ratio, where 8320 is AU of human SUMO1 immobilized onto the chip and MW ratio is the ratio of MW (Da) of SUMO1 to flavonoid. Samples were injected over SUMO1 or ubiquitin surfaces for 30s followed by 15s of dissociation phase and a wash step with 50% (v/v) DMSO solution. RU versus concentration plots were fitted in GraphPad Prism7 using classical one site binding equation to obtain approximations of K_d_ values.

### Nuclear magnetic resonance spectroscopy

All NMR experiments for 2D ^1^H-^15^N HSQC spectra were performed at 17°C on a Varian VNMR 500 MHz spectrometer equipped with a 5mm cryogenic triple resonance probe. Complex points 1024 × 128 and spectral widths of 14 × 38 ppm were used to record all the spectra. High resolution 2D ^1^H-^15^N HSQC NMR spectra were collected using 200 μM uniformly ^15^N-labeled SUMO1 treated with DMSO-d_6_ or 100–450 μM fisetin dissolved in DMSO-d_6_ using a 5 mm Wilmad 535 NMR sample tube. High resolution 2D ^1^H-^15^N HSQC NMR spectra of 100 μM uniformly ^15^N-labeled SUMO1 treated with DMSO-d_6_, quercetin or kaempferol dissolved in DMSO-d_6_ were collected using 5 mm Shigemi NMR tubes. Precipitation of quercetin and kaempferol at 400 μM precluded the use of standard 5 mm Wilmad 535-PP-7 tubes for data collection. Instead, Shigemi NMR tubes were used to collect high resolution data with 100 μM ^15^N-labeled SUMO1 and 200 μM quercetin or kaempferol dissolved in DMSO-d_6_ to obtain a 2:1 molar ratio. DMSO-d_6_ used as a cosolvent caused a slight shift (< ~0.8ppm) of resonance peaks. Therefore resonance assignments for ^15^N-labeled SUMO1 residues were compared to the DMSO control treatment and normalized by a common factor. Similar experiments were repeated for interaction studies with quercetin or kaempferol dissolved in DMSO-d_6_. The resonance peak assignments were performed using data deposited in the Biological Resonance Data Bank (BMRB) under 25576 as reference. Chemical shift changes were characterized by calculating a weighted chemical shift difference as previously described [32]. We were unable to unambiguously assign peaks for the first four amino acids of SUMO1 (MSDQ) and A72 and T41 due to peak broadening (due to slower molecular tumbling) and peak overlap in the presence of fisetin or other ligands. The chemical shift perturbation data from this work are deposited under BMRB ID 50128.

### Differential scanning fluorimetry (DSF)

Fluorescence-based thermal shift assays known as differential scanning fluorimetry (DSF) were performed following the general protocol described in [28]. In short, 20 μl reactions were prepared with 2 μM human SUMO1 or SUMO1^Δ15^ protein exchanged in DSF buffer (50 mM Tris-Cl pH 8.0, 150 mM NaCl, 10% glycerol), 10X SYPRO Orange (ThermoFisher Scientific, Waltham, MA), and 0.3 μl of DMSO control or flavonoid dissolved in DMSO. PCR plates (white non-skirted, 96-well) were sealed with optical film, centrifuged, and incubated at 4˚C for 30 min prior to analysis. Thermal scanning (20°C for 30 s, followed by ramping from 20˚C to 90˚C at 0.5˚C/15 s) was performed using a real-time PCR setup (CFX384 Touch, Biorad, Hercules, CA) with filter sets for measurement of relative fluorescence units (RFU) at 0.5°C intervals. T_m_ was calculated from the maxima of the first derivative of RFU/temperature using GraphPad Prism7. For the HSF1 denaturation experiment, recombinantly expressed and affinity purified human HSF1 was exchanged in DSF buffer. The 20 μl reaction contained 2 μM HSF1 with 0.3 μl DMSO and was either incubated on ice or treated at 60°C for 15 min followed by equilibration to room T for 60 min, prior to DSF as described above.

### Native-PAGE studies

Recombinantly purified proteins (*Saccharomyces cerevisiae* SMT3, *Saccharomyces cerevisiae* Ulp1, and human SUMO1) were incubated with flavonoids (or DMSO vehicle control) for 30 min to 1 hr at 4°C. Samples were loaded onto 4–20% or 7.5% Mini-PROTEAN TGX Precast Protein Gels (Bio-Rad, Hercules, CA) and were run positive to negative charge at 95 V for 4 hr 30 min at 4°C using a standard SDS-free loading buffer (pH 8.0) and Tris-glycine running buffer (pH 8.3). Immediately after electrophoresis, the gel was imaged to track migration of bright yellow flavonoid. Following this step, the gel was stained with Bio-Safe Coomassie (Bio-Rad, Hercules, CA) to detect the proteins.

### SDS-PAGE and immunoblotting

Proteins were separated by SDS-PAGE using 4–20% or 7.5% Mini-PROTEAN TGX Precast Protein Gels (Bio-Rad, Hercules, CA) followed by detection with Bio-Safe Coomassie Stain (Bio-Rad). For Western immunoblotting experiments, proteins in SDS-PAGE gels were transferred onto a PVDF membrane at 4°C using Mini Trans-Blot Cell (Bio-Rad), blocked with 5% nonfat milk for 1 hr at room T or overnight at 4°C and incubated with the following primary antibodies at indicated dilutions for 1 hr at room T: 1:10,000 HSF1 polyclonal antibody (Enzo Life Sciences ADI-SPA-901-F; Lausen, Switzerland), 1:4000 p53 rabbit antibody (SUMOlink SUMO-1 Kit), 1:5000 SUMO-1 rabbit antibody (SUMOlink SUMO-1 Kit). Secondary antibody incubation was performed for 1 hr at room T using 1:10,000 goat anti-rabbit IgG polyclonal antibody (Enzo Life Sciences, ADI-SAB-300). Blots were developed using SuperSignal West Femto Maximum Sensitivity Substrate (ThermoFisher Scientific, Waltham, MA) and imaged using the Azure c600 Imaging System.

### In vitro sumoylation studies

*In vitro* sumoylation experiments were performed using Active Motif SUMOlink SUMO-1 Kit (40120; Carlsbad, CA) according to the manufacturer’s instructions. Briefly, either p53 (SUMOlink kit) or HSF1, which was recombinantly expressed and purified as described above (S6 Fig in S1 Appendix) was mixed with E1 activating enzyme, E2 conjugation enzyme, SUMO1 or a conjugation-deficient SUMO1 mutant and incubated at 30°C for 3 hr prior to analysis by SDS-PAGE and immunoblotting as described above. In the SUMO1 mutant, the C-terminal glycine in the diglycine motif of the mature protein is changed to alanine [48].

## Supporting information

S1 Appendix (DOC)

S1 Raw Images (PDF)
