# Supplementary material for: Dietary flavonoid fisetin binds human SUMO1 and blocks sumoylation of p53
Source: PLoS One. 2020 Jun 12;15(6):e0234468. doi: 10.1371/journal.pone.0234468 (PMC7292393; doi:10.1371/journal.pone.0234468)
Supplement: S1 Appendix — (DOC) [file pone.0234468.s001.doc]

**Supporting Information**

Dietary flavonoid fisetin binds human SUMO1 and blocks sumoylation of p53

Vaithish Velazhahan^1,2,#a^, Przemyslaw Glaza^2^, Alvaro I. Herrera^2,#b^, Om Prakash^2^, Michal Zolkiewski^2^, Brian V. Geisbrecht^2^, Kathrin Schrick^1,3*^

^1^ Division of Biology, Kansas State University, Manhattan, Kansas, United States of America

^2^ Department of Biochemistry and Molecular Biophysics, Kansas State University, Manhattan, Kansas, United States of America

^3^ Molecular, Cellular and Developmental Biology, Kansas State University, Manhattan, Kansas, United States of America

^#a^Current Address: Medical Research Council Laboratory of Molecular Biology, Cambridge, United Kingdom

^#b^Current Address: Department of Chemistry and Biochemistry, Auburn University, Auburn, Alabama, United States of America

^*^ Corresponding author

E-mail: [kschrick@ksu.edu](mailto:kschrick@ksu.edu) (KS)

**
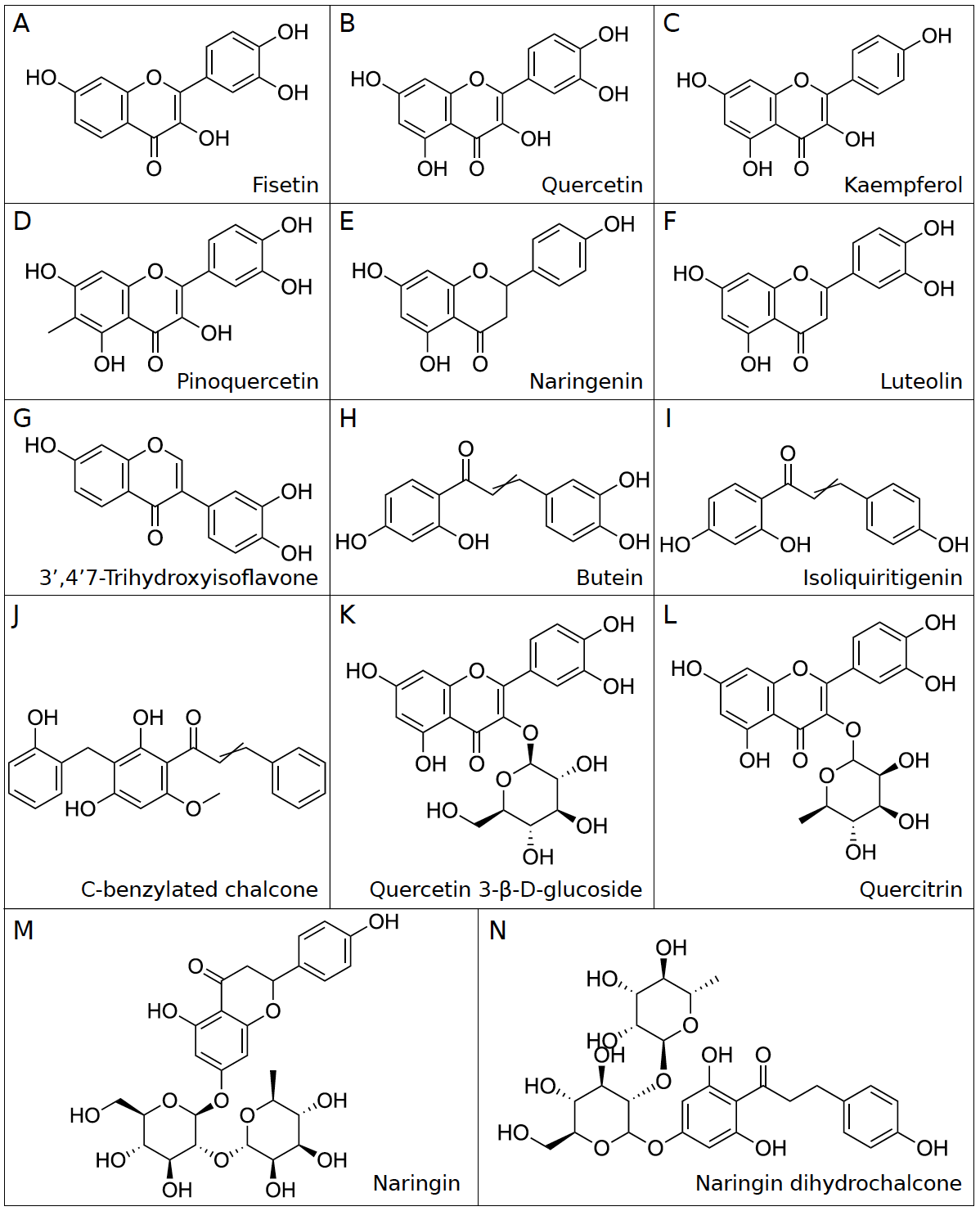
**

**S1 Fig. Structures of flavonoids in this study.**

The 14 flavonoids listed in Table 1 are grouped according to structural similarity. (A-D) Flavonols: Fisetin, Quercetin, Kaempferol, and Pinoquercetin. (E) Flavanone: Naringenin. (F-G) Flavones: Luteolin and 3’,4’ 7-Trihydroxyisoflavone. (H-J) Chalcones: Butein, Isoliquiritigenin, and C-benzylated chalcone. (K-N) Glycosylated flavonoids: Quercetin 3-β-D glucoside, Quercitrin, Naringin, and Naringin dihydrochalcone.

**
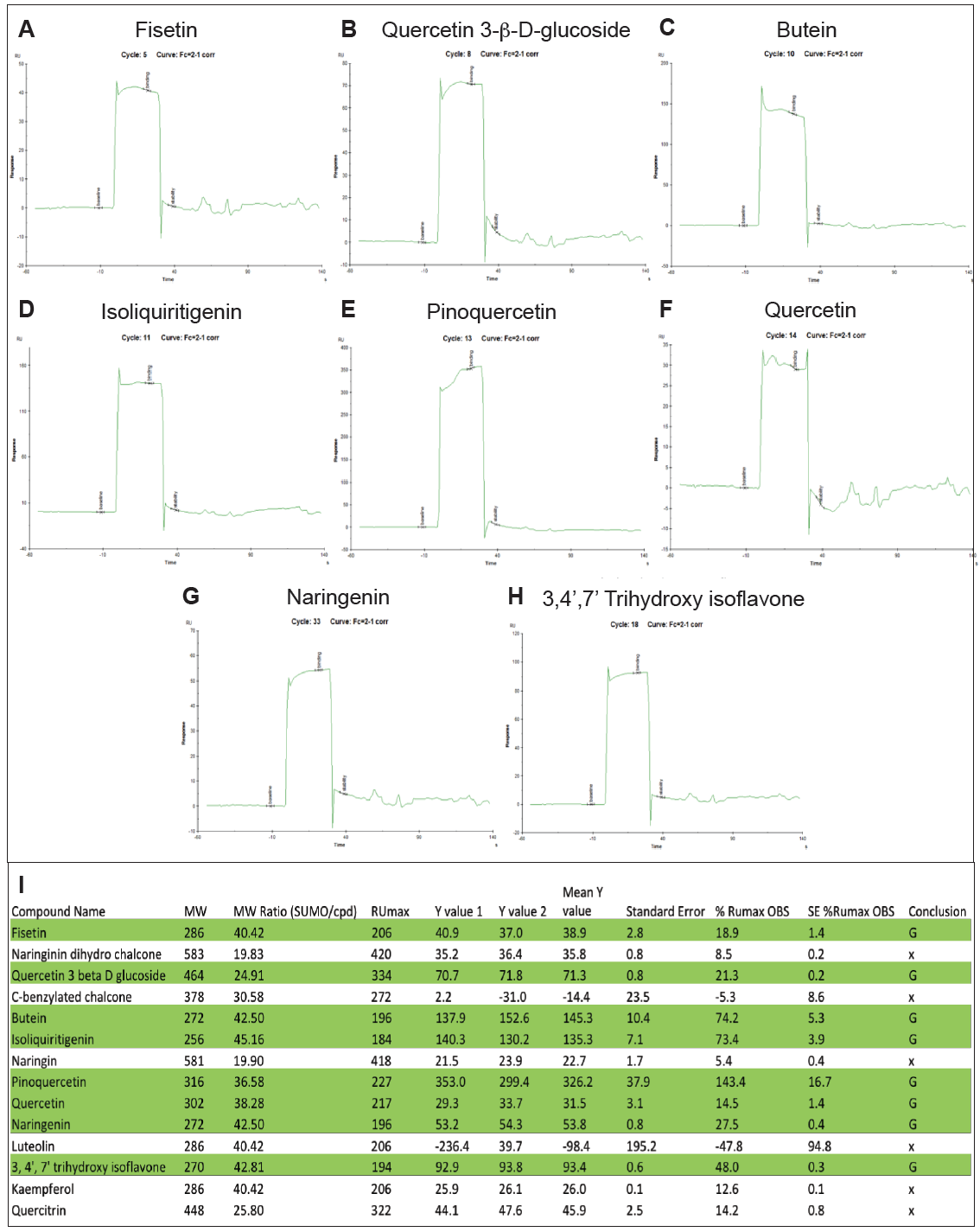
**

**S2 Fig**. **Surface plasmon resonance (SPR) primary screen results.**

Sensograms of positive hits from the SPR primary screen are shown as follows: (A) Fisetin, (B) Quercetin 3-β-D-glucoside, (C) Butein, (D) Isoliquiritigenin, (E) Pinoquercetin, (F) Quercetin, (G) Naringenin, and (H) 3’,4’,7-Trihydroxyisoflavone. (I) Raw data of SPR primary screen performed in duplicate (Yvalue1 and Yvalue2). Out of 14 flavonoids, eight (A-H) were selected for secondary screening based on a %(RU_max_)obs of ~15% (or greater), indicated by “G*”* under “Conclusion”.

**
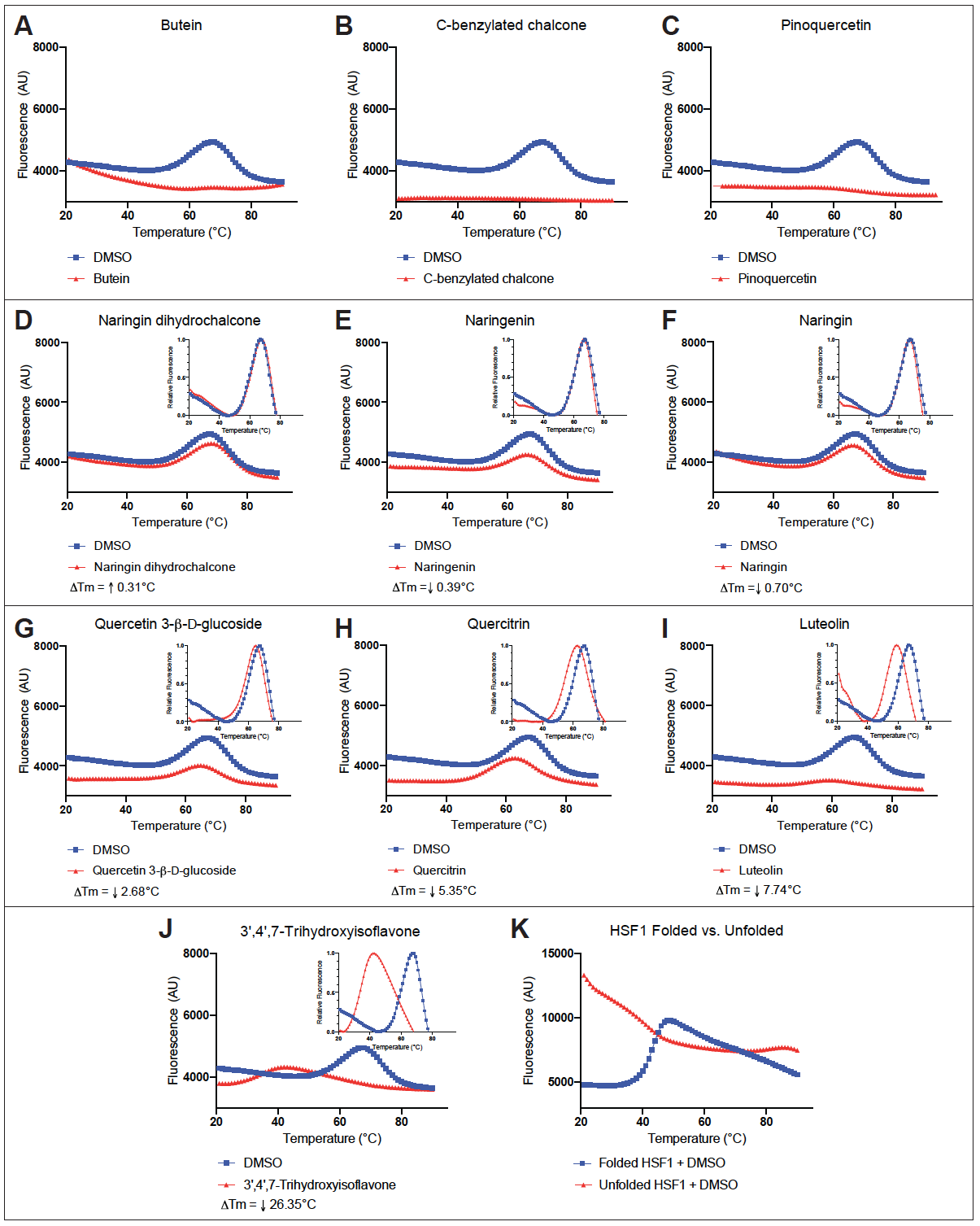
**

**S3 Fig**. **Summary of differential scanning fluorimetry (DSF) data described in Fig 4 and Table 1.**

Human SUMO1 protein (2 µM) was mixed with flavonoid (300 µM in DMSO) versus DMSO control. DSF thermograms are shown for (A) Butein, (B) C-benzylated chalcone, (C) Pinoquercetin, (D) Naringin dihydrochalcone, (E) Naringenin, (F) Naringin, (G) Quercetin 3-β-D-glucoside, (H) Quercitrin, (I) Luteolin, (J) 3’,4’,7-Trihydroxyisoflavone. (D-J) Normalized thermograms are shown in insets and change in melting temperature (ΔT_m_) relative to DMSO control is indicated. (K) HSF1 unfolding: 2 µM human HSF1 treated with DMSO was heated at 60°C for 15 min and allowed to equilibrate to room T for 1 hr. DSF thermogram from unfolded HSF1 resembles DSF thermogram of human SUMO1 treated with 300 µM fisetin, quercetin, or isoliquiritigenin (Fig 4A-C) suggesting that these flavonoids induce partial unfolding of SUMO1. DSF of unfolded HSF1 was performed in two replicates and DSF of folded HSF1 was performed in five replicates.

**
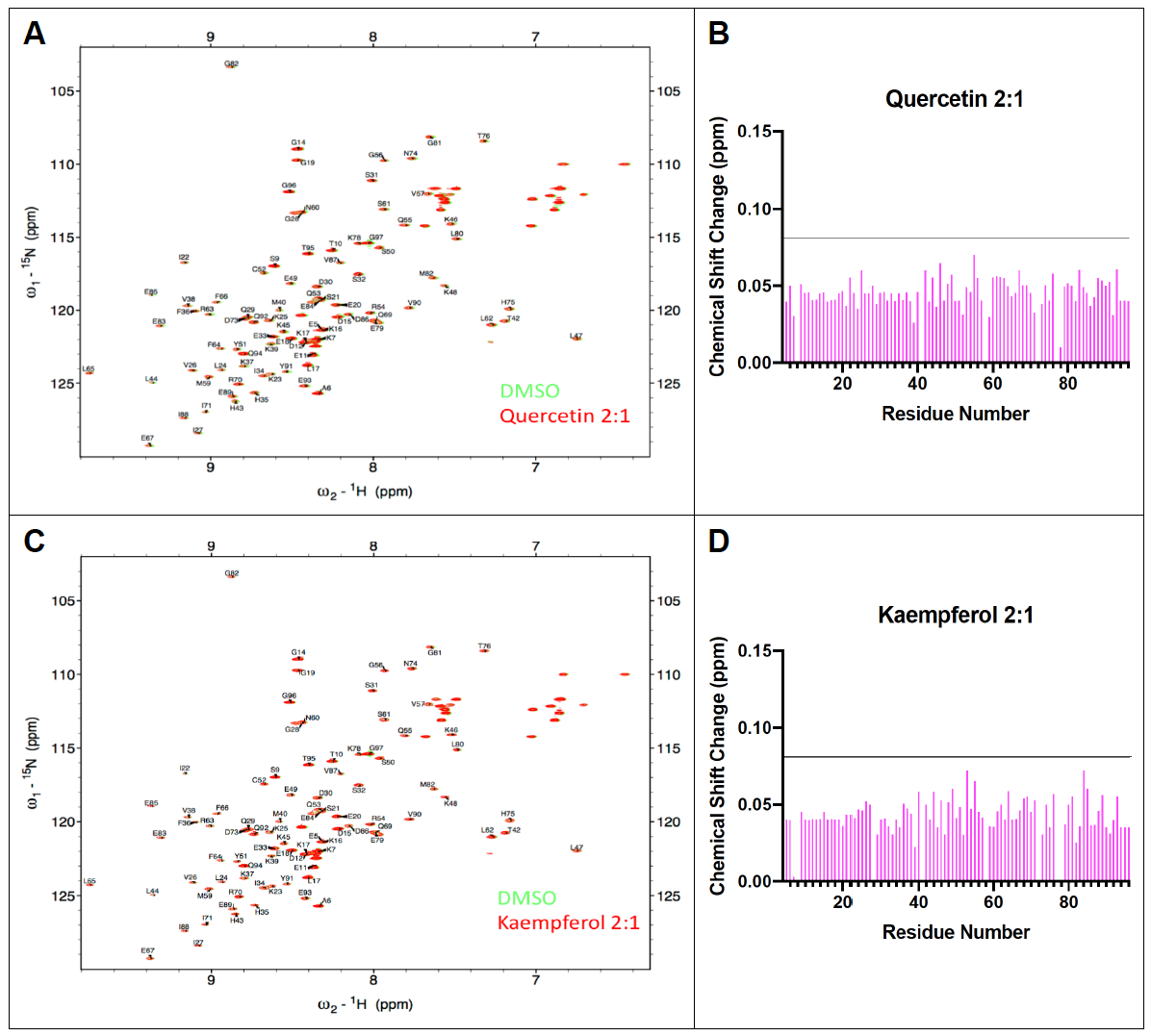
**

**S4 Fig**. **^1^H-^15^N HSQC analyses of SUMO1 with quercetin or kaempferol fail to show a chemical shift perturbation.**

^1^H-^15^N HSQC spectrum of SUMO1 upon treatment with DMSO vehicle control versus a 2:1 molar ratio of (A,B) quercetin or (C,D) kaempferol to protein. Peak assignments and HSQC spectra were rendered using NMRFAM-Sparky [1]. Perturbation analyses of HSQC spectra failed to detect a chemical shift above the cut-off of 0.08. Chemical shift change was calculated as previously described [2]. See Fig 6.

**
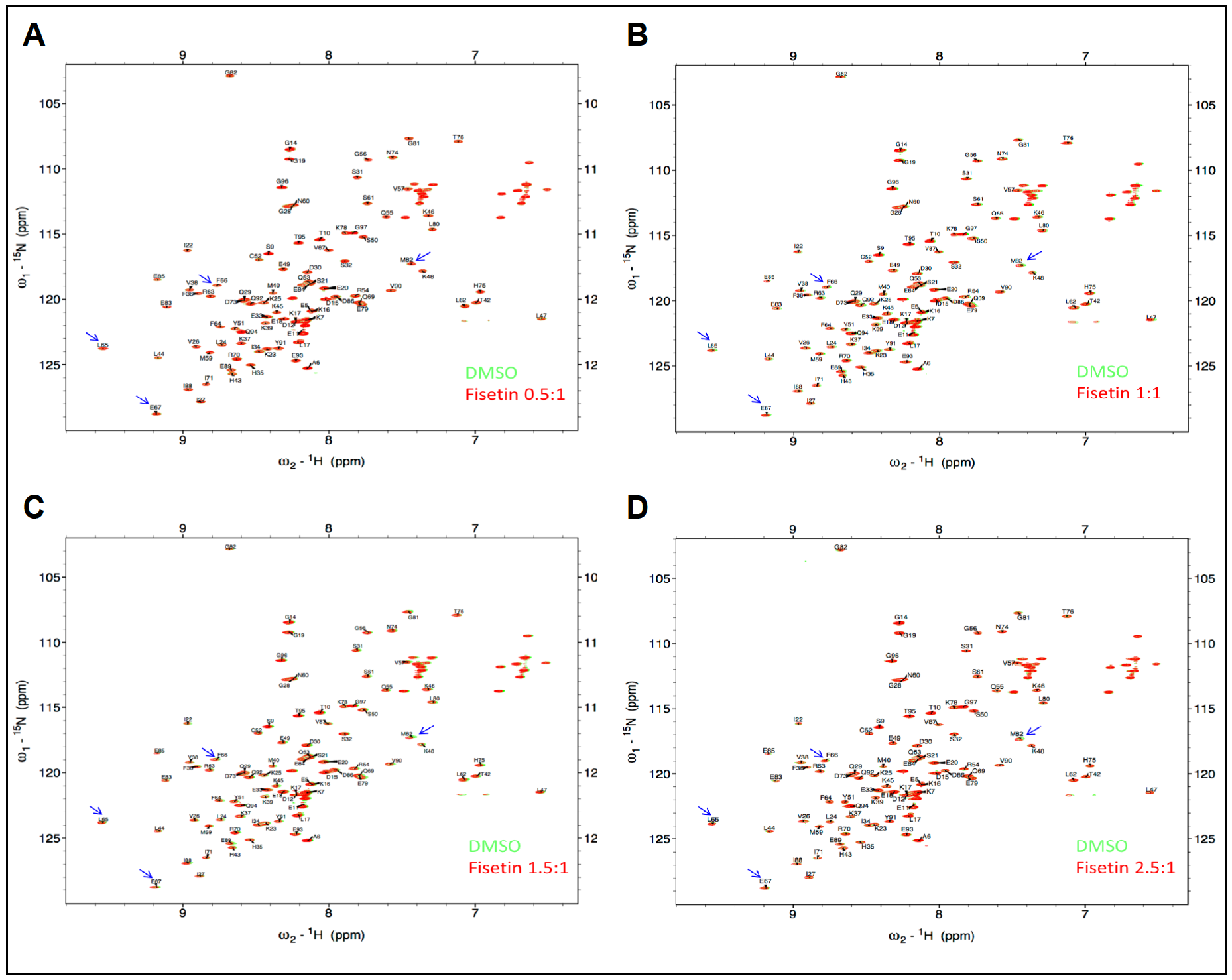
**

**S5 Fig.** **^1^H-^15^N HSQC titration analyses of SUMO1 with fisetin.**

^1^H-^15^N HSQC spectra were obtained at increasing molar ratios of fisetin to SUMO1. SUMO1 protein (200 µM) remained constant while the concentration of fisetin was varied as follows: **(A)** 100 µM (0.5:1 ratio), **(B)** 200 µM (1:1 ratio), **(C)** 300 µM (1.5:1 ratio), and **(D)** 450 µM (2.5:1 ratio). HSQC spectrum obtained at 400 µM fisetin (2:1 ratio) is shown in Fig 6A.

**
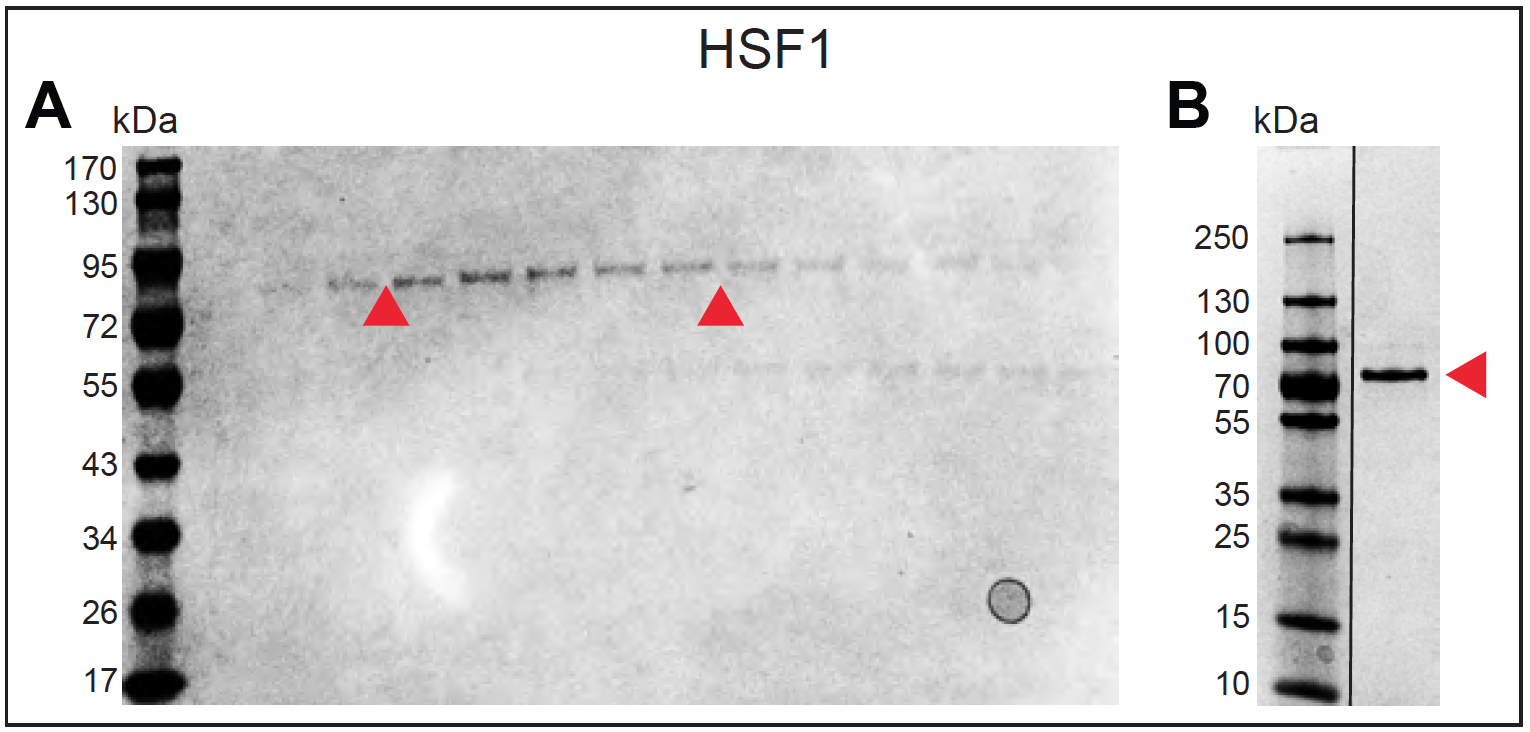
**

**S6 Fig. Purification of recombinantly expressed human HSF1 protein.**

(A) SDS-PAGE of HSF1 fractions purified by size exclusion chromatography. Red arrowheads delineate the fractions that were pooled. (B) SDS-PAGE of purified and pooled HSF1 protein (red arrowhead). Molecular weight marker and HSF1 protein were run on the same gel. The line between the two lanes indicates removal of one lane between the marker and HSF1.


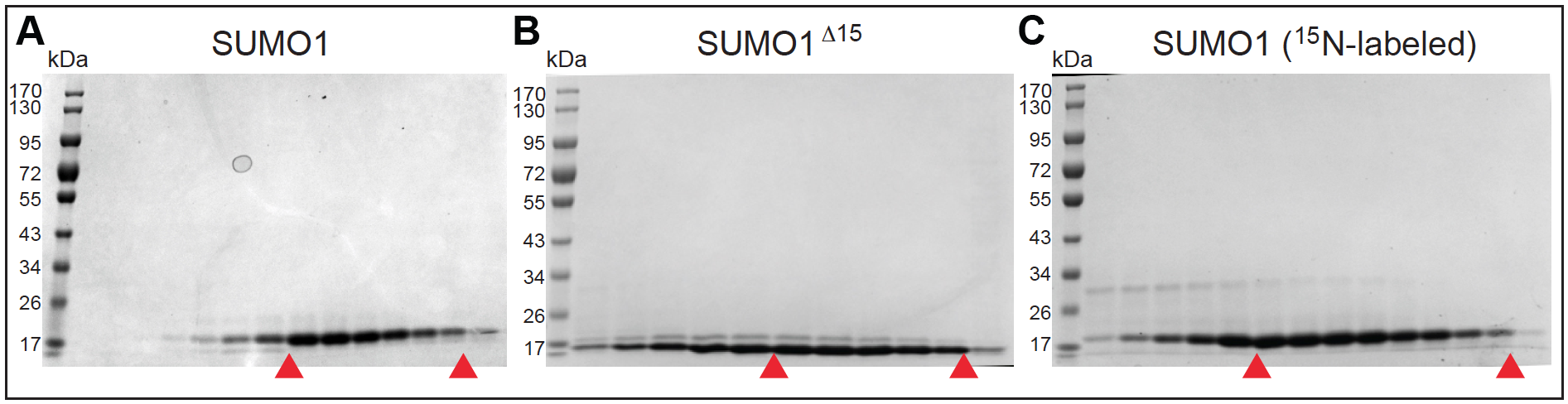


**S7 Fig. Purification of recombinantly expressed human SUMO1 protein.**

(A-C) SDS-PAGE analysis of SUMO fractions purified by size exclusion chromatography. Red arrowheads delineate the fractions that were pooled to prepare protein samples. (A) Wild-type human SUMO1, (B) Mutant SUMO1^Δ15^ in which the N-terminal 15 amino acids of SUMO1 are deleted, and (C) ^15^N-labeled SUMO1 that was prepared for the NMR chemical shift perturbation studies.

**Supporting Information References**

1. Lee W, Tonelli M, Markley JL. NMRFAM-SPARKY: enhanced software for biomolecular NMR spectroscopy. Bioinformatics. 2015;31(8):1325-7.

2. Grzesiek S, Bax A, Clore GM, Gronenborn AM, Hu JS, Kaufman J, et al. The solution structure of HIV-1 Nef reveals an unexpected fold and permits delineation of the binding surface for the SH3 domain of Hck tyrosine protein kinase. Nature Structural Biology. 1996;3(4):340-5.
