# Supplementary material for: Dietary flavonoid fisetin binds human SUMO1 and blocks sumoylation of p53
Source: PLoS One. 2020 Jun 12;15(6):e0234468. doi: 10.1371/journal.pone.0234468 (PMC7292393; doi:10.1371/journal.pone.0234468)

S1_raw_images

Fig 1A-F

Fig 7A-D

Supporting Information S6 Fig A and B

Dietary flavonoid fisetin binds human SUMO1 and blocks sumoylation of p53

Vaithish Velazhahan[1,2,#a], Przemyslaw Glaza[2], Alvaro I. Herrera[2,#b], Om Prakash[2], Michal Zolkiewski[2], Brian V. Geisbrecht[2], Kathrin Schrick[1,3*]

[1] Division of Biology, Kansas State University, Manhattan, Kansas, United States of America

[2] Department of Biochemistry and Molecular Biophysics, Kansas State University, Manhattan, Kansas, United States of America

[3] Molecular, Cellular and Developmental Biology, Kansas State University, Manhattan, Kansas, United States of America

[#a]Current Address: Medical Research Council Laboratory of Molecular Biology, Cambridge, United Kingdom

[#b]Current Address: Department of Chemistry and Biochemistry, Auburn University, Auburn, Alabama, United States of America

[*] Corresponding author

E-mail: kschrick@ksu.edu (KS)

Fig 1A. Coomassie stained Native-PAGE showing a protein mixture containing SMT3 protein incubated with no ligand (NL), DMSO (-), fisetin (+F), or quercetin (+Q) for 30 min on ice prior to running the gel at 95 V at 4°C. The bovine serum albumin (BSA) size marker is shown on the left. Monomer (66 kDa), dimer and trimer of BSA are indicated. The mobility-shifted band in the "+F" lane was excised and analyzed by mass spectrometry. Peptides that were over-represented in this sample identified the SMT3 protein as the predominant component (data not shown).

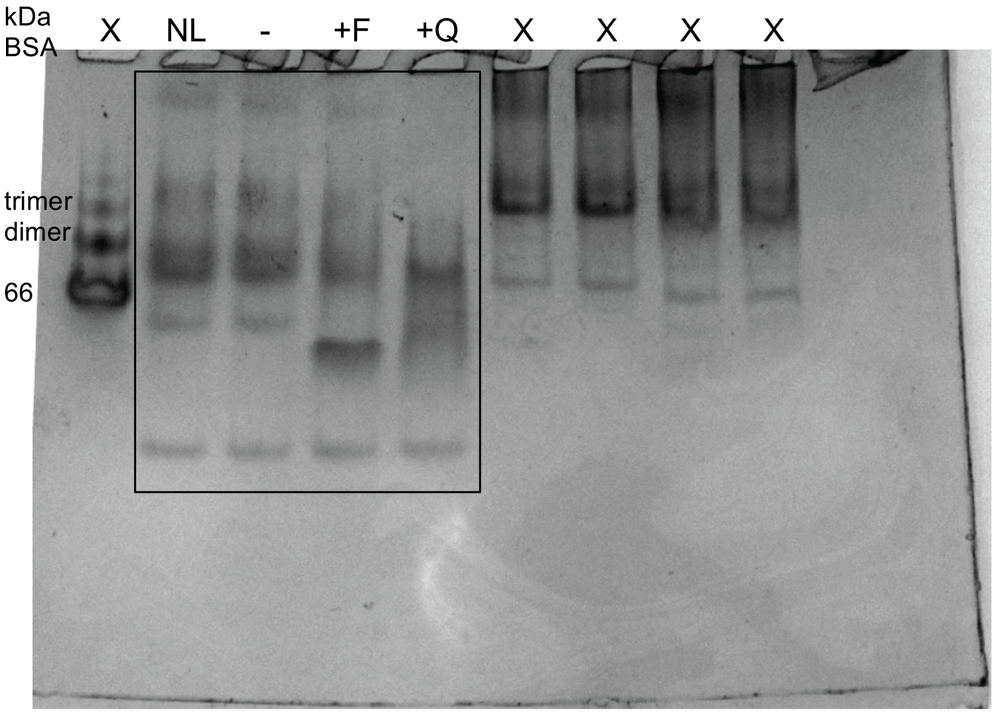

Fig. 1B. Coomassie stained Native-PAGE showing SMT3 protein (2.75 μM) that was incubated for 30 min on ice with increasing concentrations of fisetin (2.34 to 300 μM), prior to running the gel at 95V at 4°C. DMSO (-) control is shown on the left.

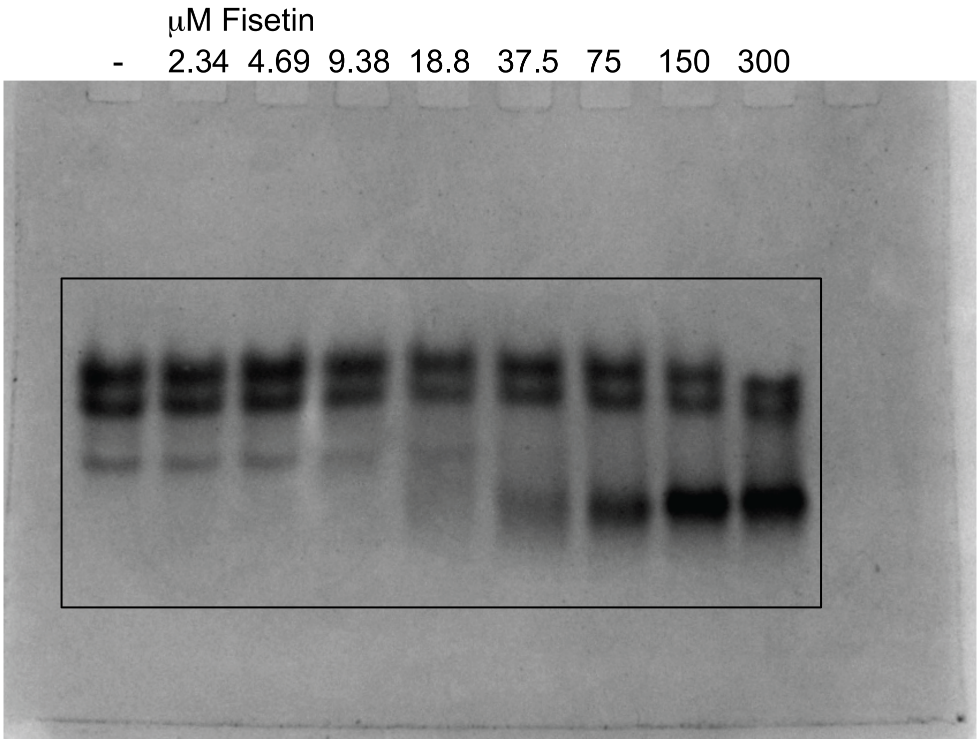

Fig 1C. (left) Coomassie stained Native-PAGE showing the Ulp1 protein (~1-2 μM) incubated in the presence of DMSO (-) or 300 μM fisetin (+F) for 30 min on ice prior to running the gel at 95V at 4°C. The BSA size marker is shown on the left. Monomer (66 kDa), dimer and trimer of BSA are indicated.

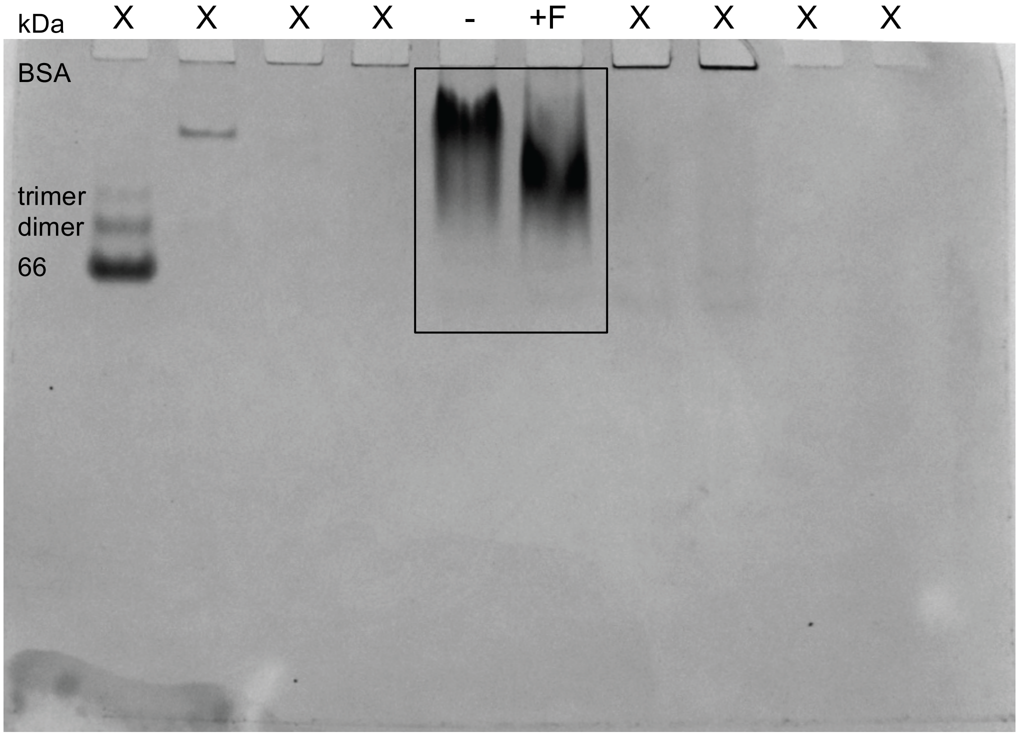

Fig. 1C (right). Native-PAGE showing the Ulp1 protein (2 μM) incubated for 30 min on ice with increasing concentrations of fisetin (2.34 to 300 μM), prior to running the gel at 95V at 4°C. The BSA size marker is shown on the left. Monomer (66 kDa), dimer and trimer of BSA are indicated.

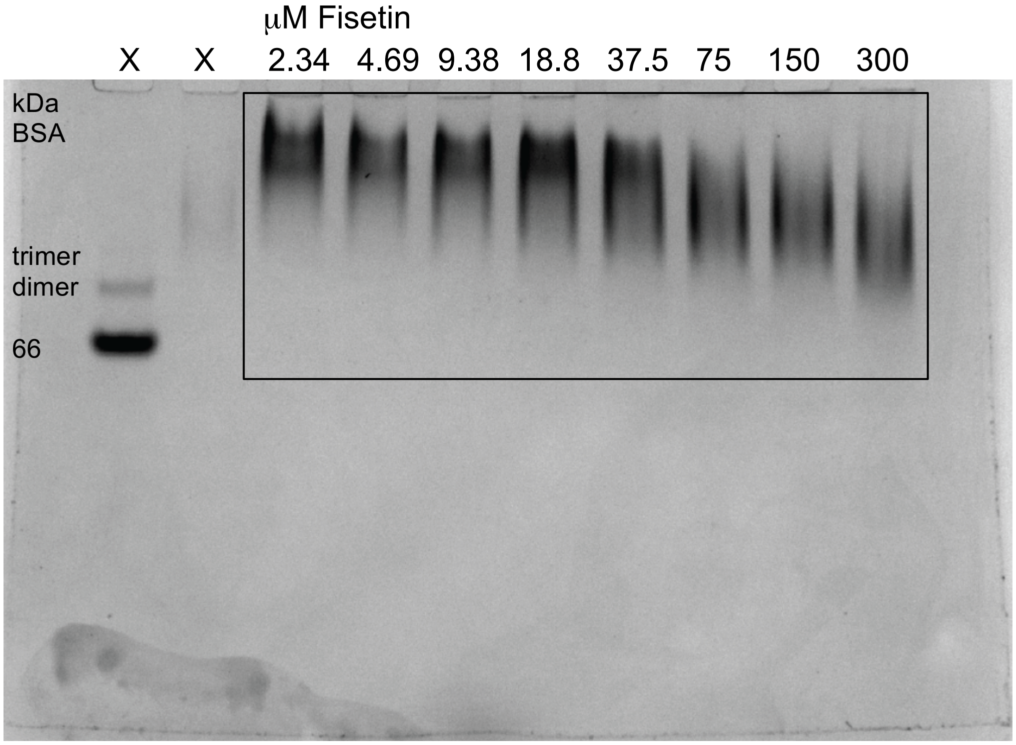

Fig. 1D. Native-PAGE showing BSA protein incubated with no ligand (NL), DMSO (-), fisetin (+F), or quercetin (+Q) for 30 min on ice prior to running the gel at 95 V at 4°C. The BSA size marker is shown on the left. Monomer (66 kDa), dimer and trimer of BSA are indicated.

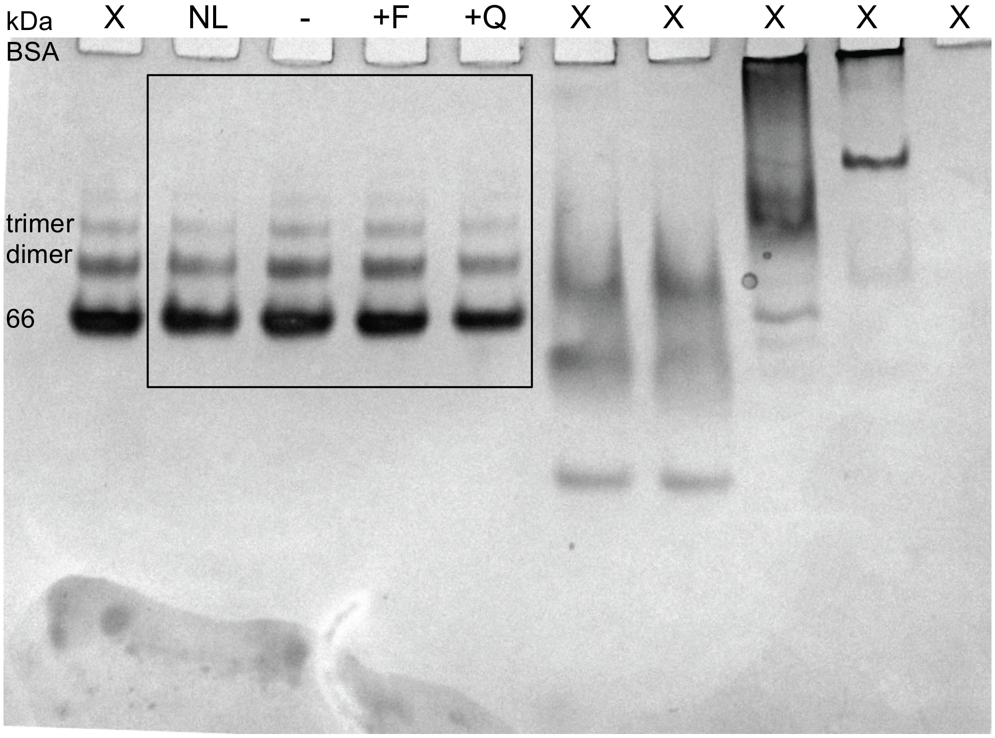

Fig. 1E. Left: Coomassie stained Native-PAGE showing Ulp1 and SMT3 proteins incubated with DMSO (-) or fisetin (+F) for 30 min on ice prior to running the gel at 95 V at 4°C. **Right:** Matching gel image prior to Coomassie staining. Fisetin is visible by its bright yellow color. The gel-shifted form of Ulp1 or SMT3 upon fisetin treatment matches the yellow fisetin band. The no protein (NP) control lane indicates a reaction without protein or fisetin; The only fisetin (OF) control contained only fisetin. The BSA size marker is shown on the left. Monomer (66 kDa), dimer and trimer of BSA are indicated.

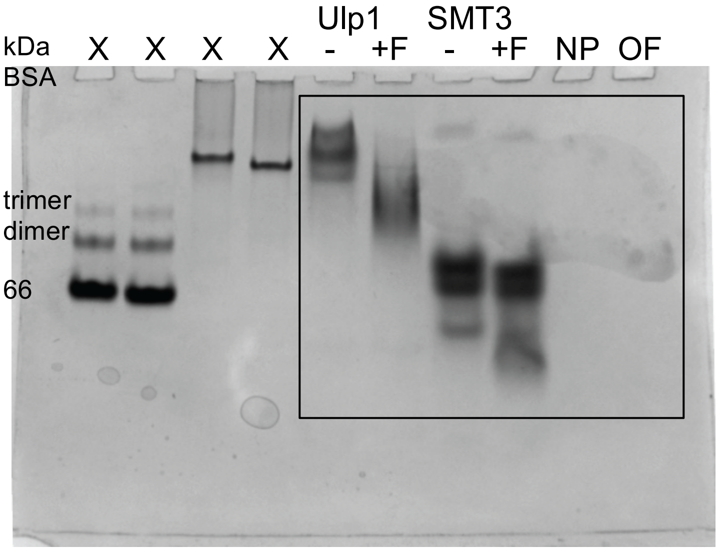

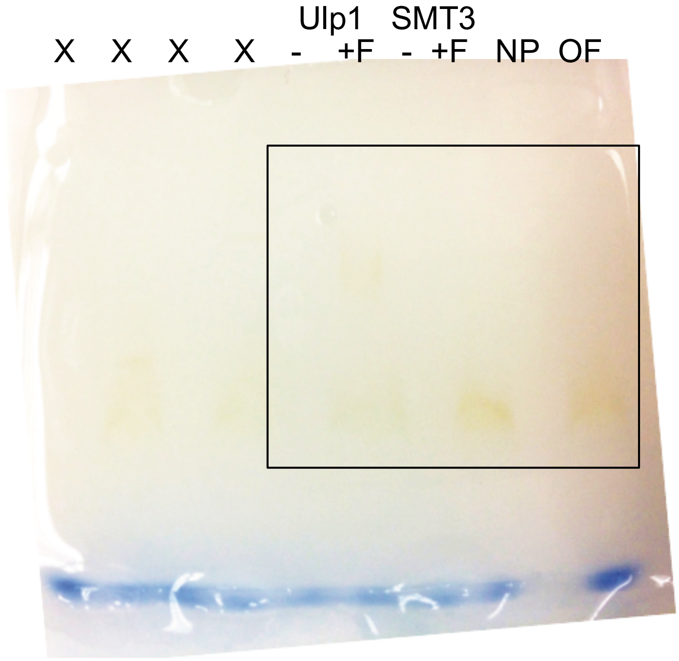

Fig. 1F. Coomassie stained Native-PAGE showing the human SUMO1 protein incubated with luteolin (L), DMSO (-), fisetin (+F), or quercetin (+Q) for 30 min on ice prior to running the gel at 95 V at 4°C. The NativeMark protein standard (Thermofisher Scientific, Waltham, MA) is shown on the left.

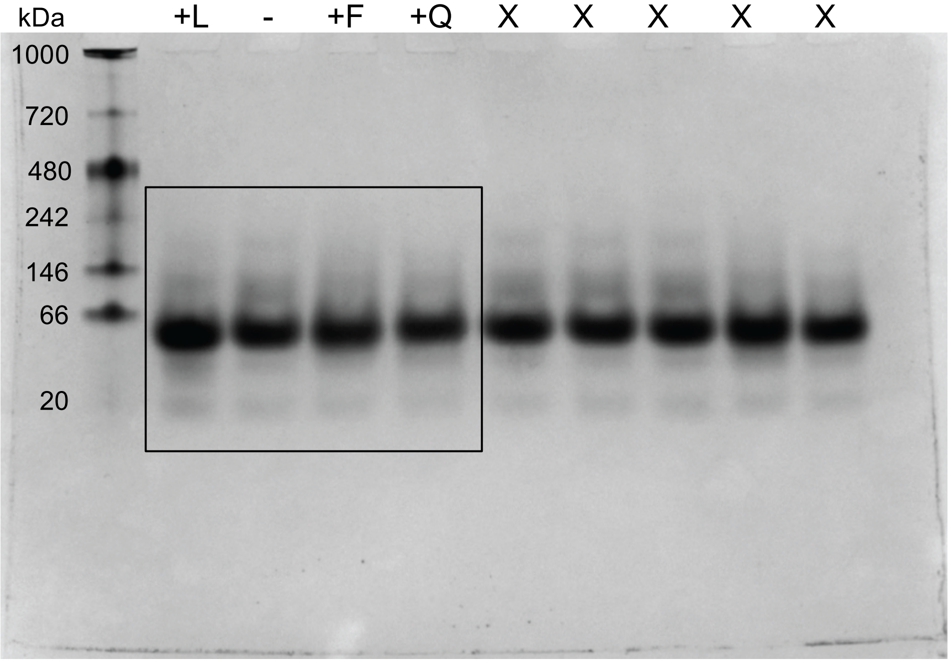

Fig. 7A. *In vitro* sumoylation of tumor suppressor protein p53 in the absence of (-, DMSO control) or presence of fisetin (+F, 300 µM dissolved in DMSO). Western blot was probed with a SUMO1 antibody (1:5000 dilution) from the SUMOlink SUMO-1 kit (Active Motif, Carlsbad, CA). A SUMO1 conjugation-deficient mutant (mt) served as a negative control. A no substrate (NS) control allowed detection of E1 and E2-SUMO conjugates with the SUMO1 antibody. In **Fig. 7A**, the two boxed images are placed adjacent to each other.

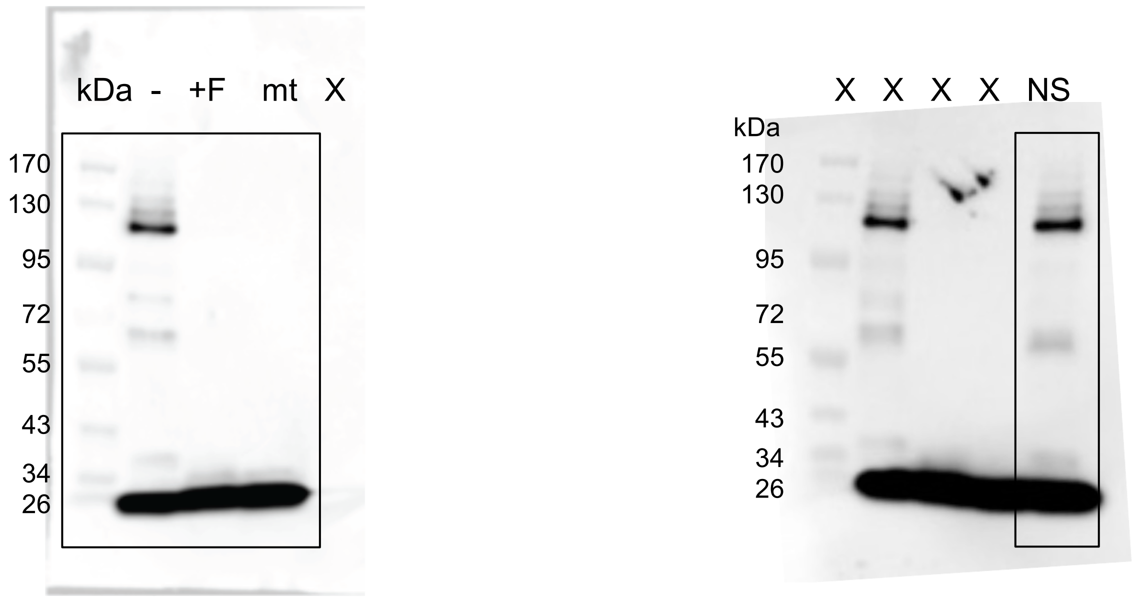

Fig. 7B. *In vitro* sumoylation of tumor suppressor protein p53 in the absence of (-, DMSO control) or presence of fisetin (+F, 300 µM dissolved in DMSO). Western blot was probed with p53 antibody (1:7500 dilution) from the SUMOlink SUMO-1 kit (Active Motif, Carlsbad, CA). A SUMO1 conjugation deficient mutant (mt) served as a negative control. A no substrate (NS) control allowed detection of E1 and E2-SUMO conjugates with the SUMO1 antibody. In **Fig. 7B**, the two boxed images are placed adjacent to each other.

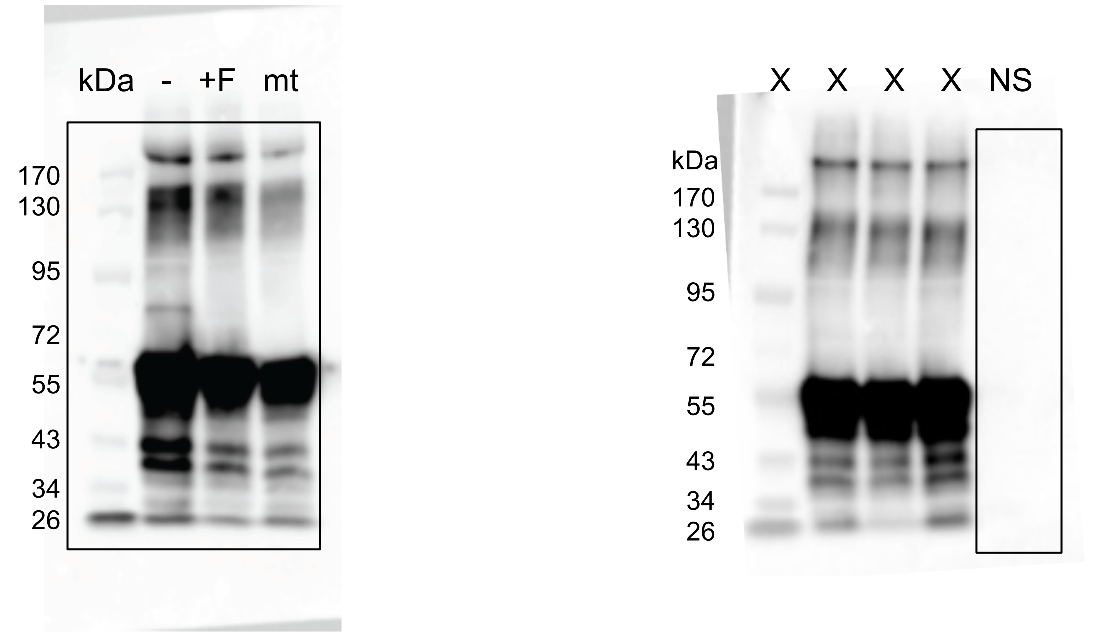

Fig. 7C. *In vitro* sumoylation of Heat Shock Factor 1 (HSF1) protein in the absence of (-, DMSO control) or presence of fisetin (+F, 300 μM dissolved in DMSO). Western blot was probed with SUMO1 antibody (1:4000 dilution) from the SUMOlink SUMO-1 kit (Active Motif, Carlsbad, CA). A SUMO1 conjugation-deficient mutant (mt) served as a negative control. A no substrate (NS) control allowed detection of E1 and E2-SUMO conjugates with the SUMO1 antibody.

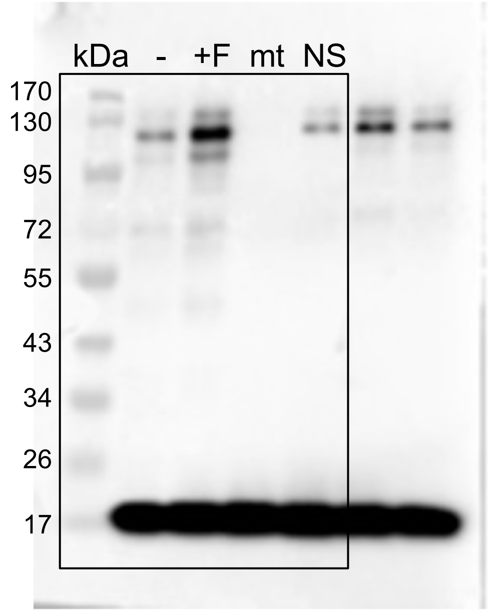

Fig. 7D. *In vitro* sumoylation of Heat Shock Factor 1 (HSF1) protein in the absence of (-, DMSO control) or presence of fisetin (+F, 300 μM dissolved in DMSO). Western blot was probed with a HSF1 polyclonal antibody (1:10,000 dilution) (Enzo Life Sciences, ADI-SAB-300, Lausen, Switzerland). A SUMO1 conjugation-deficient mutant (mt) served as a negative control. A no substrate (NS) control allows detection of E1 and E2-SUMO conjugates with the SUMO1 antibody.

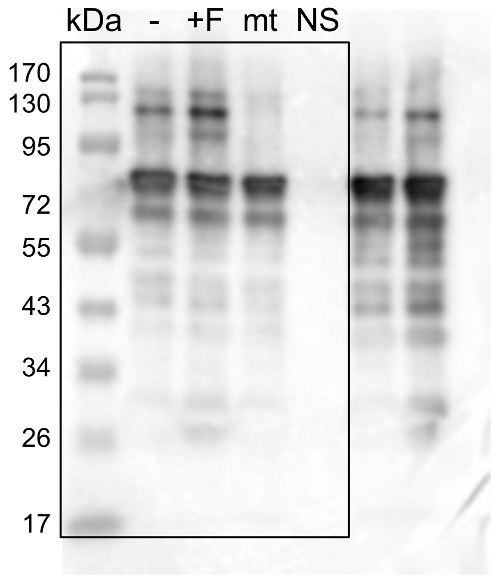

Supporting Information

S6 Fig. A. Purification of recombinantly expressed HSF1. Coomassie stained SDS-PAGE of HSF1 fractions (1-14) purified by size exclusion chromatography. Fractions 4-8 were collected for downstream analyses.

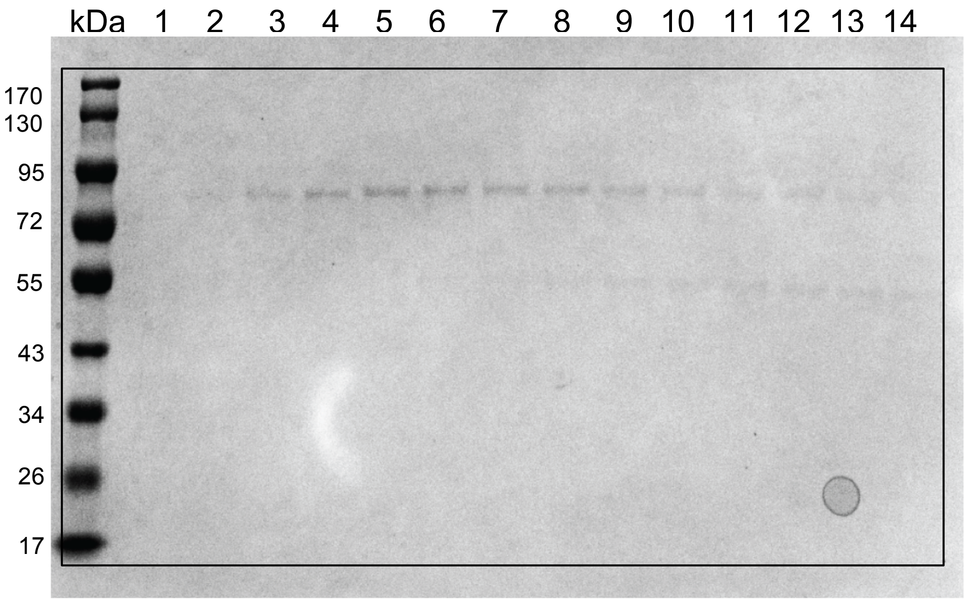

S6 Fig. B. Purification of recombinantly expressed HSF1. Coomassie stained SDS-PAGE of purified and pooled HSF1 protein. Molecular weight marker and HSF1 protein were run on the same gel. The figure in the Supporting Information placed the two indicated lanes adjacent to each other.

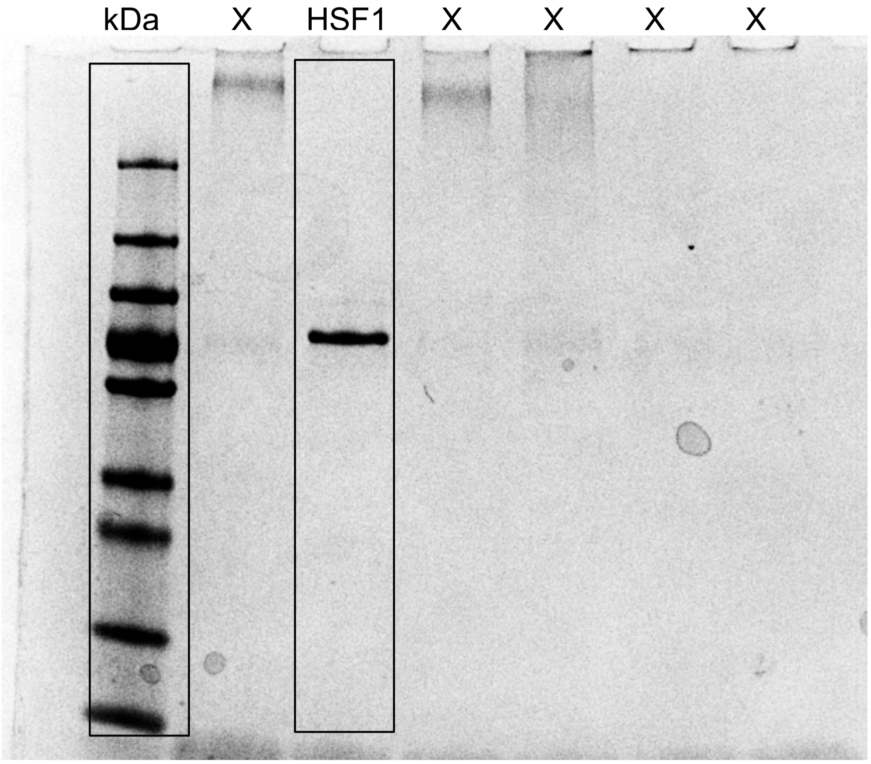

S7 Fig. A. Purification of recombinantly expressed human SUMO1 protein. Coomassie stained SDS-PAGE of wild-type SUMO1 fractions (1-14) purified by size exclusion chromatography. Fractions 8-13 were collected for downstream analyses.

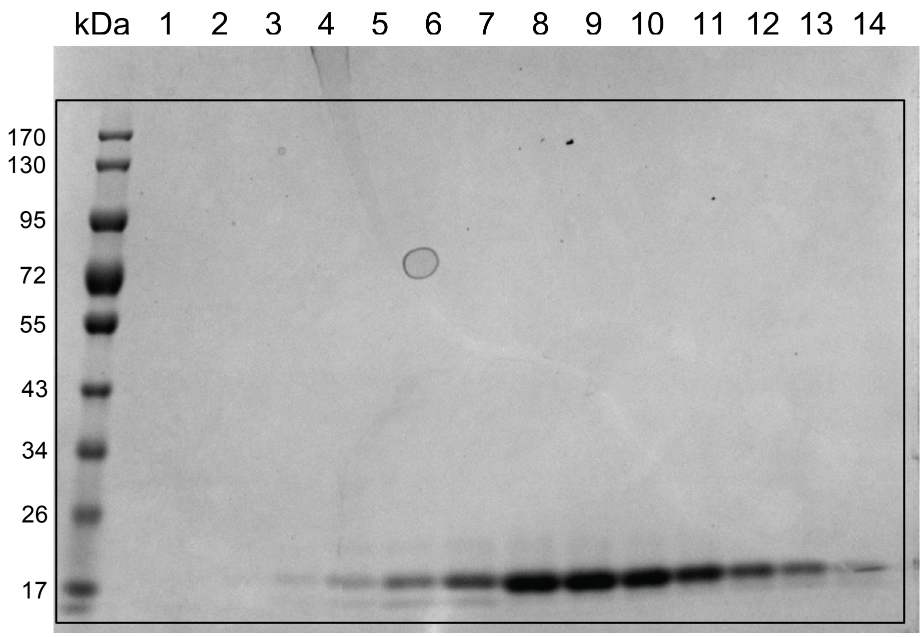

S7 Fig. B. Purification of recombinantly expressed mutant SUMO1$^{\Delta 15}$ protein. Coomassie stained SDS-PAGE of mutant SUMO1$^{\Delta 15}$ fractions (1-11) purified by size exclusion chromatography. In mutant SUMO1$^{\Delta 15}$, the N-terminal 15 amino acids of SUMO1 are deleted. Fractions 6-10 were collected for downstream analyses.

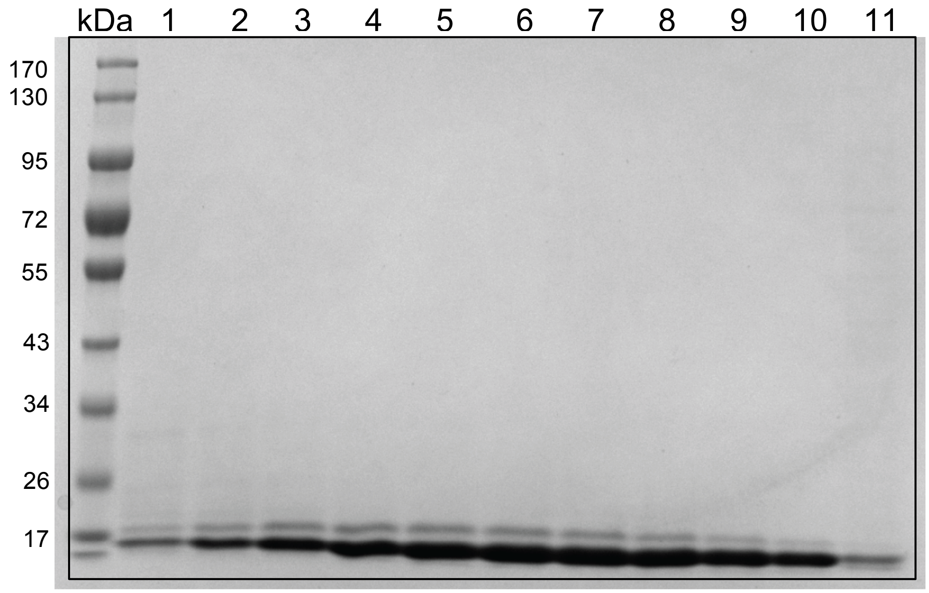

S7 Fig. C. Purification of recombinantly expressed human SUMO1 protein. Coomassie stained SDS-PAGE of ^{15}N-labeled SUMO1 fractions (1-14) purified by size exclusion chromatography. Fractions 6-13 were collected for the NMR chemical shift perturbation studies.

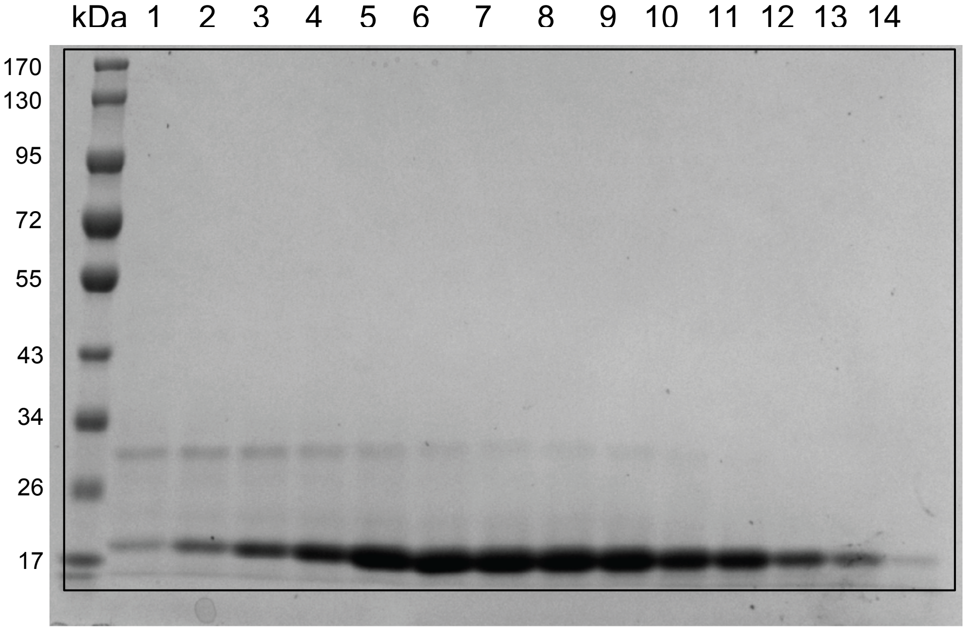

Supplement: S1 Raw Images — (PDF) [file pone.0234468.s002.pdf]
